# Integrative analysis of transcriptome and lipidome reveals fructose pro-steatosis mechanism in goose fatty liver

**DOI:** 10.3389/fnut.2022.1052600

**Published:** 2023-01-10

**Authors:** Rongxue Wei, Chunchun Han, Shouhai Wei, Yongqiang Teng, Liang Li, Hehe Liu, Shengqiang Hu, Bo Kang, Hengyong Xu

**Affiliations:** ^1^Farm Animal Genetic Resources Exploration and Innovation Key Laboratory of Sichuan Province, Sichuan Agricultural University, Chengdu, China; ^2^Key Laboratory of Livestock and Poultry Multi-omics, Ministry of Agriculture and Rural Affairs, College of Animal Science and Technology, Sichuan Agricultural University, Chengdu, China

**Keywords:** fructose, goose fatty liver, peripheral adipose tissues, transcriptome, lipidome

## Abstract

To further explore the fructose pro-steatosis mechanism, we performed an integrative analysis of liver transcriptome and lipidome as well as peripheral adipose tissues transcriptome analysis using samples collected from geese overfed with maize flour (control group) and geese overfed with maize flour supplemented with 10% fructose (treatment group). Overfeeding period of the treatment group was significantly shorter than that of the control group (*p* < 0.05). Dietary supplementation with 10% fructose induced more severe steatosis in goose liver. Compared with the control group, the treatment group had lower in ceramide levels (*p* < 0.05). The key differentially expressed genes (DEGs) (control group vs. treatment group) involved in liver fatty acid biosynthesis and steroid biosynthesis were downregulated. The conjoint analysis between DEGs and different lipids showed that fatty acid biosynthesis and steroid biosynthesis were the highest impact score pathways. In conclusion, fructose expedites goose liver lipid accumulation maximization during overfeeding.

## Introduction

It has long been speculated that fructose consumption plays an important role in the development of non-alcoholic fatty liver disease (NAFLD). Many reports found that the NAFLD formation mechanism was mediated by fructose (1, 2). Endoplasmic reticulum stress (ERS) induces insulin resistance (IR) and NAFLD in mammals (3). Yu et al. (4) reported that X-Box Binding Protein-1 (XBP-1), one mark of ERS, partially mediated high-fructose-induced fat deposition *via de novo* lipogenesis augmentation in HepG2 cells. High-fructose foods promoted fatty acid synthesis in hepatocytes, resulting in IR and fat accumulation in the liver (5). “Gut-liver axis” theory (6) reveals the relationship between the liver and gut and has attracted lots of attention in studying these diseases. Some researchers reported that NAFLD induced by fructose was closely related to the intestinal barrier (7). After a large amount of fructose enters the organism, it will cause endoplasmic reticulum stress and intestinal inflammation, resulting in intestinal barrier damage, bacterial escape, and endotoxemia. Increased pro-inflammatory cytokines will induce liver lipid synthesis and deposition and then promote the occurrence of non-alcoholic steatohepatitis (NASH) (8). After overfeeding, the overfed goose or duck received plenty of high-energy carbohydrates; the liver of the overfed goose or duck increased 5–10 fold in 2 weeks and was accompanied by severe hepatic steatosis. This unique genetic mechanism is was used to produce *foie gras*. A previous study found that there were similarities between overfed geese and humans or mammals with NAFLD in serum enzyme activity changes and liver lipid deposition mechanisms (9). As a consequence, goose fatty liver can be used as an excellent model of non-alcoholic fatty liver for biomedical research (10). Our preliminary research showed that fructose effectively promoted goose liver fat accumulation in overfeeding (11). However, the fructose pro-steatosis mechanism needs further exploration.

Our previous research work suggested that overfeeding through diet supplementation with fructose promoted intestinal digestion and absorption capacity and increased the enrichment of *Lactobacillus* in the intestine (12). Goose fatty liver formation is mainly because of the imbalance between fatty acid synthesis, β-oxidation, lipid synthesis, and lipid transportation in the liver. Lipidomics is an important branch of metabolomics and has played an important role in the study of liver disease. Lipidomics, as a widely used analytical technique to analyze lipid metabolism, is helpful to search for NAFLD-related biomarkers. Numerous liver diseases have been found to cause changes in plasma lyso-phosphatidylcholines (LPC) levels, making LPC a potential biomarker for NAFLD. The ratio of phosphatidylcholines (PC) to phosphatidylethanolamines (PE) is a determinant of cell membrane integrity and a predictor of NAFLD (13). However, the fructose pro-steatosis mechanism has not been elucidated from the goose liver lipidome perspective. The interaction between the liver and peripheral adipose tissue plays an important role in the process of NAFLD development. Adipose tissue release effector molecules, for example adipokines, mediate lipid metabolism and contribute to promoting fat deposition in the liver (14). In addition, adipose tissue, especially visceral adipose tissue, contains multiple cell populations which secrete adipocyte factor and insulin-like growth factor (IGF), macrophages, and other immune cells which play an important role in the process of lipotoxic hepatic disease development (15). However, what role peripheral adipose tissues play is unclear in the process where fructose promotes overfed goose liver lipid deposition. In the current study, goose liver transcriptome analysis and lipidome analysis were integrated; in addition, the interaction between the peripheral adipose tissues and the liver was investigated, which will provide new insights into the fructose pro-steatosis mechanism in overfed goose liver. *Foie gras*, the fatty liver of overfed ducks or geese, is the highest-value product in waterfowl production. However, *foie gras* production has encountered bottlenecks because of the heavy labor intensity and low feed conversion ratio, as well as animal welfare concerns. Our preliminary research showed that fructose strongly promotes fat accumulation in overfed goose liver (11), which suggested that dietary supplementation with fructose may be a potential approach to improving the efficiency of *foie gras* production and animal welfare (for example, reducing the overfeeding intensity or shortening the overfeeding time). Thereby, not only will understanding the fructose pro-steatosis mechanism in goose fatty liver formation provide a reference to preventing the non-alcoholic steatohepatitis induced by fructose in humans but also a scientific basis to ensure animal welfare.

## Materials and methods

### Ethics statement

The experiment was conducted in accordance with the Institutional Animal Care and Use Committee (IACUC) of Sichuan Agricultural University (Permit No. DKY-B20141401). This experiment was carried out at the Experimental Farm for Waterfowl Breeding of Sichuan Agricultural University (Ya'an, Sichuan, China).

### Birds and experiment design and sampling

A total of 60 healthy 90-day-old male Tianfu meat geese were randomly separated into a control group and a treatment group. The ganders of the control group were overfed with raw (uncooked) maize flour (60% dry matter + 40% water). The ganders in the treatment group were overfed with maize flour supplemented with 10% fructose. During overfeeding, the daily feed intake gradually increased. The daily feed intake reached 1,800–2,000 g (five meals per day) on the 6th day. Overfeeding lasted 18 days. The overfed ganders had free access to water, and the feed formula of the diet for the experiment is provided in Supplementary Table S1. The routine husbandry management, overfeeding procedure, and diet regimes were carried out through the overfeeding experiments. Our previous preliminary study demonstrated that fructose had a better promotion of fat accumulation during goose fatty liver formation. However, the mortality rate was too high (11). To further explore the fructose pro-steatosis mechanism in goose fatty liver formation, we redesigned the study. To gain better goose fatty liver performance and avoid high mortality rates, overfed ganders were slaughtered in time during the overfeeding period in the current study. When the overfed ganders were lethargic, fat, and unable to walk around, the ganders were slaughtered in time. The remaining ganders were slaughtered together on the 18th day of overfeeding. After slaughter, the liver tissue, the subcutaneous fat tissue, the abdominal fat tissue, and the intestine-mesentery fat tissue were collected and weighed. Each liver was separated into three parts: the first part was kept at −20 °C for water content and crude fat determination; the second part was collected for histomorphology determination, which was washed with ice-cold 0.9% NaCl saline and fixed with 4% formaldehyde-phosphate buffer; the third part was frozen at −80 °C for transcriptome and lipidome determination. Subcutaneous fat tissue, abdominal fat tissue, and intestine-mesentery fat tissue were kept at −80 °C for transcriptome determination.

### Crude fat determination and histology examination by Oil Red O staining

The freeze-drying method was used to detect the water content of the liver tissue. Approximately 0.5 g (m_1_) ground liver tissue was wrapped with filter paper (marked with pencil), and the total weight (m_2_) was taken and put in a vacuum freeze dryer (Thermo Fisher Scientific, USA). After 72 h, the total weight (m_3_) was weighed, and the water content was calculated by the formula: water content (%) = m_2_-m_3_/${\text{m}}_{1}^{*}$100%. Crude fat was determined using the Soxhlet extraction method. After freeze-drying, the dry liver tissue (m_4_) was wrapped with filter paper (the total weight was m_5_) and put in a Soxhlet extractor (diethyl ether extraction 24 h, water bath heating at 50°C). The total weight (m_6_) was measured after extraction: crude fat content (%) = (m_5_-m_6_/m_4_) ^*^100%. The determination was repeated three times.

The methods of Oil Red O staining were performed according to the manufacturer's instructions. (1) Dehydration: the formalin-fixed liver tissue was placed in 10% sucrose solution, removed after the tissue sank to the bottom and placed in 20% sucrose solution, and removed after the tissue sank to the bottom and placed in 30% sucrose solution, and then removed and frozen quickly after the tissue sank to the bottom. (2) Cryosection preparation: dehydrated liver tissue was embedded with an OCT embedding agent and then sliced (the microtome was prepared at −20 °C), and the thickness of the section was 10–15 μm. (3) Washing: after drying, the section was slightly washed with 50% ethanol. (4) Staining: the washed section was stained with Oil Red O solution (Sigma, USA) for 8–10 min; after color separation with 50% ethanol, the stained section was washed with tap water, re-stained with hematoxylin violet (purchased from Nanjing Jiancheng Technology Co., LTD.) and then sealed with glycerin gelatin. Each slice was examined by microscope photography system (Olympus Optical, Tokyo, Japan) at 200 × magnifications.

### Transcriptome analysis

A total amount of 1 μg RNA per sample was used as the RNA preparations for sequencing. The construction of sequencing libraries was performed according to the manufacturer instructions of the NEBNext UltraTM RNA Library Prep Kit. The purification of library fragments was performed *via* the AMPure XP system (Beckman Coulter, Beverly, USA). Three biological replicates for each tissue (liver tissue, subcutaneous fat tissue, abdominal fat tissue, and intestine-mesentery fat tissue). Total RNA from frozen samples (approximately 100 mg) were extracted with RNeasy Mini Kit (QIAGEN, Germany). RNA integrity was checked by Agilent Bioanalyzer 2100 (Agilent Technologies, CA, USA). All libraries were sequenced by the Illumina NexSeq500 platform, which was performed by Suzhou PANOMIX Biomedical Tech Co., LTD (Jiangsu, China). After sequencing, the image files were transformed to generate raw data of FASTQ *via* the software of the sequencing platform. The raw data of each sample was calculated separately, which contained sample name, Q30, percentage of fuzzy bases, and Q20(%) and Q30(%) (Supplementary Table S1). The sequencing raw data should be further filtered. The basic information of data filtering is shown in Supplementary Table S2. After finishing the examination involved in base mass distribution, base content distribution, and the average quality distribution of reads, sequencing assembly was performed. For transcriptome sequencing projects without reference genomes, we used Trinity software to assemble clean reads. After sequencing assembly, the transcript sequence file in FASTA format can be obtained for subsequent analysis. The longest transcript extracted was the unigene. Gene function annotation was based on KEGG and GO (gene ontology).

### Lipidome analysis

Six liver samples that came from the control group and six liver samples that came from the treatment group were provided for lipidome detection. Lipid extraction was performed as follows: (1) 100 mg samples were transferred into a 2 ml Eppendorf tube, and 750 μl −20°C chloroform methanol solution (2:1) was added; (2) after grinding, the samples were kept in the ice for 40 min; (3) after adding 190 μl ddH_2_O and vortex-mixed for 30 s, the samples were kept in the ice for 10 min; (4) after centrifugation (12,000 rpm, 5 min), 300 μl lower layer fluid was transferred into a new Eppendorf tube; (5) 500 μl chloroform–methanol mixed solution was added and mixed for 30 s at −20°C; (6) after centrifugation (12,000 rpm, 5 min), 400 μl lower layer fluid was transferred into the same Eppendorf tube; (7) after concentration, 200 μl isopropanol was added and vortexed; (8) after centrifugation (12,000 rpm, 5 min), the supernatant was obtained and filtered through 0.22 μm membrane; and (9) 20 μl supernatant from each sample was transferred for quality control, and the rest of the supernatant was used for liquid chromatography–mass spectrometry (LC-MS) (Thermo, USA) detection.

The Thermo Vanquish system was used for chromatographic separation. Chromatographic conditions were performed as follows: the chromatograph column used was ACQUITY UPLC^®^ BEH C18 (100 × 2.1 mm, 1.7 μm, Waters); column temperature was maintained at 50°C. Mobile phases contained A_2_ and B_2_, A_2_ - acetonitrile: water = 60:40 (0.1% formic acid+10 mM ammonium formate), B_2_ - isopropanol: acetonitrile = 90:10 (0.1% formic acid+10 mM ammonium formate); flow rate was 0.25 ml/min; injection volume was 2 μl. Gradient elution program was executed as follows(v/v): 0–5 min, 70-57% A_2_; 5–5.1 min, 57%-50% A_2_; 5.1–14 min, 50–30% A_2_; 14–14.1 min, 30% A_2_; 14.1–21 min, 30–1% A_2_; 21–24 min, 1% A_2_; 24–24.1 min, 1–70% A_2_; 24.1–28 min, 70% A_2_.

Thermo Q Exactive Focus mass spectrometer is equipped with positive and negative ion ionization modes: positive ion spray voltage is 3.50 kV, negative ionization–ionization voltage of 2.50 kV was used for mass analysis. Sheath gas was 30 arb, and auxiliary gas was 10 arb. The capillary temperature was 325°C, full scan was performed at a resolution of 35,000, and the mass range was m/z 150–2,000. Data collection was conducted by taking the HCD scan (collision voltage was 30 eV), and the unnecessary MS/MS information was removed the by dynamic exclusion method. Lipidome determination and analysis were performed by Suzhou PANOMIX Biomedical Tech Co., LTD (Jiangsu, China).

### Data analysis

GraphPad Prism 5.0 software (GraphPad Prism Software, Inc.) was used to perform T-test and visualization for all slaughter performance data. All slaughter performance data were presented as means ± standard deviation (SD). We considered *p* < 0.05 as statistically significant. DEseq was used to perform the gene differential expression analysis between the control group and the treatment group (adjusted *p*-values were used to control the false discovery rate). The genes with an adjusted *p* < 0.05 were considered differentially expressed genes (DEGs). Gene Ontology (GO) analysis and KEGG analysis involved in DEGs were implemented by the GOseq R packages and KOBAS software, respectively. The LipidSearch software (V4) was applied to study the lipidome profile difference between the control group and the treatment group. The significantly different lipids were screened from the orthogonal partial least squares-discriminant analysis (OPLS-DA) model (VIP > 1.0 and *p* < 0.05). Lipid data are shown as mean ± SD. We considered a *p* < 0.05 as statistically significant. Cluster analysis, correlation analysis, principal component analysis (PCA), partial least squares-discriminant analysis (PLS-DA), and OPLS-DA analyses were conducted to reveal the lipidome profile difference between the two groups. Related KEGG pathways of the lipids were searched by the Kyoto Encyclopedia of Genes and Genomes (KEGG). MetaboAnalyst 5.0, using Joint Pathway Analysis (https://www.metaboanalyst.ca/), was applied to perform the conjoint analysis between liver transcriptome and lipidome.

## Results

### Fructose pro-steatosis in liver tissue

**Figure 1:**
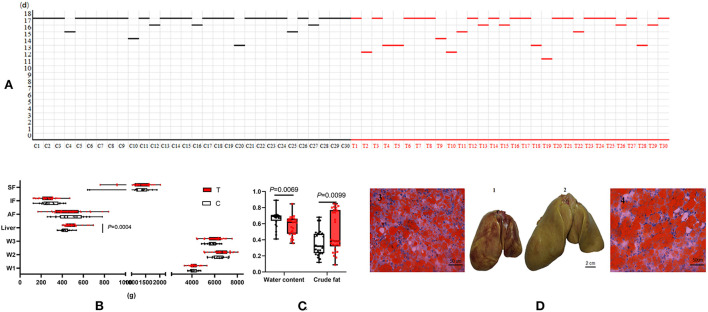
Dietary supplementation of 10% fructose influenced overfed goose liver lipid accumulation. **(A)** Comparison of overfeeding time between control group and treatment group (the line represents the point of slaughter in time during an 18-day-long overfeeding period); **(B)** data between control group and treatment group (*n* = 30) after slaughter; **(C)** comparison of liver water content and crude fat content between control group and treatment group (*n* = 30); **(D)** comparison of livers and liver tissue sections between control group and treatment group (*n* = 3). (1) Goose liver of control group after 18 days of overfeeding. (2) Goose liver of treatment group after 18 days of overfeeding. (3) Goose liver tissue section of control group after 18 days overfeeding (200 ×). (4) Goose liver tissue section of treatment group after 18 days overfeeding (200 ×). C, control group; T, treatment group; W1, body weight before overfeeding; W2, body weight after overfeeding; W3, slaughter weight; SF, subcutaneous fat; AF, abdominal fat; IF, intestine-mesentery fat.

The data from this study showed that the overfeeding period of the treatment group (geese overfed with maize flour supplemented with 10% fructose) was significantly shorter than that of the control group (geese only overfed with maize flour) (16.53 ± 1.925 vs. 17.50 ± 1.042 d) (*p* < 0.05) (Figure 1A). The liver weight and liver lipid content of the treatment group were higher than those of the control group. The liver yellowness of the treatment group was higher than that of the control group (Figure 1C). Liver tissue slices stained with Oil Red O also showed that more lipid droplets were deposited in the hepatocytes of the treatment group (Figure 1D). Dietary supplementation with 10% fructose more effectively induced lipid deposition in the livers of overfed geese. However, there was no significant difference between the control group and treatment group in peripheral adipose tissue weight (*p* > 0.05) (Figure 1B).

### Transcriptome analysis of goose fatty liver and peripheral adipose tissue

To illustrate the lipid deposition difference between the control group and treatment group, cDNA libraries from the livers and the peripheral adipose tissues were constructed and sequenced. After eliminating 3′ adapter sequences (cut adapter), duplicated sequences, and low-quality reads (<Q20), about 93% clean reads were obtained from the liver tissue, and more than 92% clean reads were obtained from peripheral adipose tissues (Supplementary Table S2). After *de novo* assembly of the clean reads, the number of unigenes was 91,101 in the liver; the total number of unigenes was 2,15,474 in peripheral adipose tissues (Supplementary Table S3). The gene expression level was represented by RPKM, and the RPKM value for each gene was calculated. The DEGs were identified between the control group and treatment group (DEGseq software, |log2FoldChange| > 1 and *p* < 0.05). MA plot depicting the DEGs is presented in Supplementary Figure S1. A total of 944 (376 downregulated), 1,344 (754 downregulated), 1,551 (846 downregulated), and 745 DEGs (434 downregulated) were identified in liver, abdominal fat tissue, intestinal-mesentery fat tissue, and subcutaneous fat tissue, respectively. Different tissues of DEGs involved in lipid metabolism, cell cycle, and anti-inflammation are shown in Supplementary Table S4. In the liver, the gene expression levels of key DEGs involved in fatty acid synthesis (*FASN, ELOVL6, SREBP1, fabF, FADS2, HMGCR, SCD, acs, HSD17B7*, and *ERG25*) were downregulated, the gene expression level of liver lipoprotein lipase (*LPL*) was upregulated; the gene expression levels of key DEGs involved in the cell cycle (*PDL1* and *P21*) were upregulated; the gene expression levels of key DEGs involved in cell apoptosis and inflammatory response (*CASP1, CMPK2, IL-9, IL-36, IL-20RB, PRF, HF1, NFKBID, BAT (CFH)*, and *LAG3*) were downregulated. In abdominal fat tissue, the gene expression levels of *SCD* and *GLUT4* were downregulated. In subcutaneous fat tissue, the gene expression levels of *G6PC, dgkA, CCNA, glpK*, and *ACSL* were upregulated. In intestine-mesentery fat tissue, the gene expression levels of *ACSL, CD44, CD36, lip, glpK, LPL, CD99, LRP1*, and *dgkA* were upregulated.

**Figure 2:**
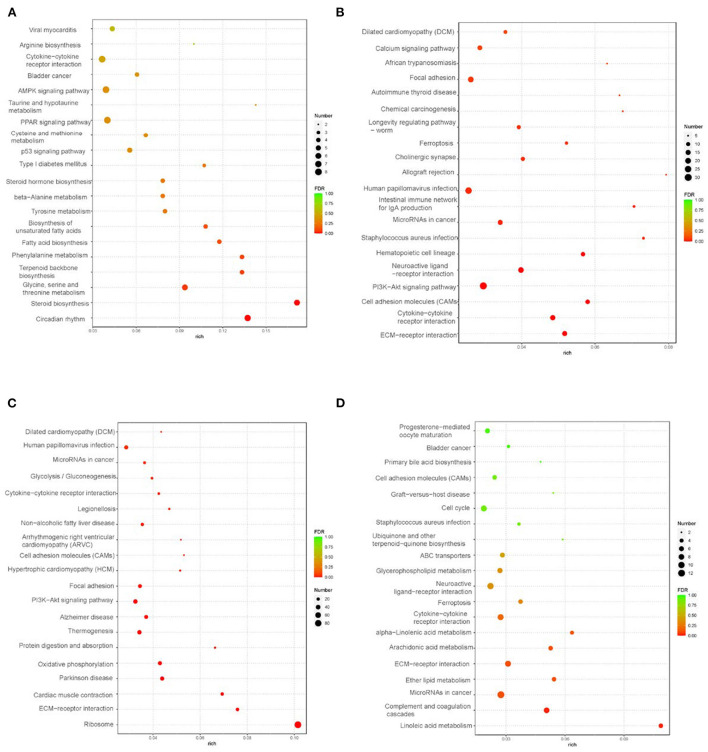
KEEG analysis in liver and peripheral adipose tissues (control vs. treatment) (*n* = 3). **(A)** KEGG analysis of liver tissue; **(B)** KEGG analysis of abdominal fat tissue; **(C)** KEGG analysis of intestine-mesentery fat tissue; **(D)** KEGG analysis of subcutaneous fat tissue.

GO pathway analysis was performed to explore functional enrichment. In liver tissue (Supplementary Figure S2), the functional groups were mainly enriched in the biological process of lipid metabolism. In abdominal adipose tissue (Supplementary Figure S3), the biological process functional groups were mainly enriched in cell migration, locomotion, motility, and localization. In intestinal-mesentery fat tissue (Supplementary Figure S4), the biological process functional groups were mainly enriched in the peptide biosynthetic process and amide metabolic process. In subcutaneous fat tissue (Supplementary Figure S5), the biological process functional groups were mainly enriched in the cellular developmental process and cell differentiation. KEGG pathway analysis of the DEGs is shown in Figure 2. In liver tissue, the top five significantly enriched KEGG pathways were circadian rhythm; steroid biosynthesis; glycine, serine, and threonine metabolism; terpenoid backbone biosynthesis; and phenylalanine metabolism (Figure 2A). In abdominal adipose tissue, the top five KEGG pathways were enriched in the “Environmental Information Processing” category, which contained ECM-receptor interaction, cytokine–cytokine receptor interaction, cell adhesion molecules (CAMs), PI3K-Akt signaling pathway, and neuroactive ligand-receptor interaction (Figure 2B). In intestinal-mesentery fat tissue, the highest enrichment pathways were the genetic information processing-category-ribosome pathway (Figure 2C). In subcutaneous fat tissue, the top five KEGG pathways were enriched in five categories, respectively: cellular processes-ferroptosis; environmental information processing-ECM receptor interaction; human diseases-microRNAs in cancer; metabolism-linoleic acid metabolism; and organismal systems-complement and coagulation cascades (Figure 2D).

### Lipidome analysis of goose fatty liver

**Figure 3:**
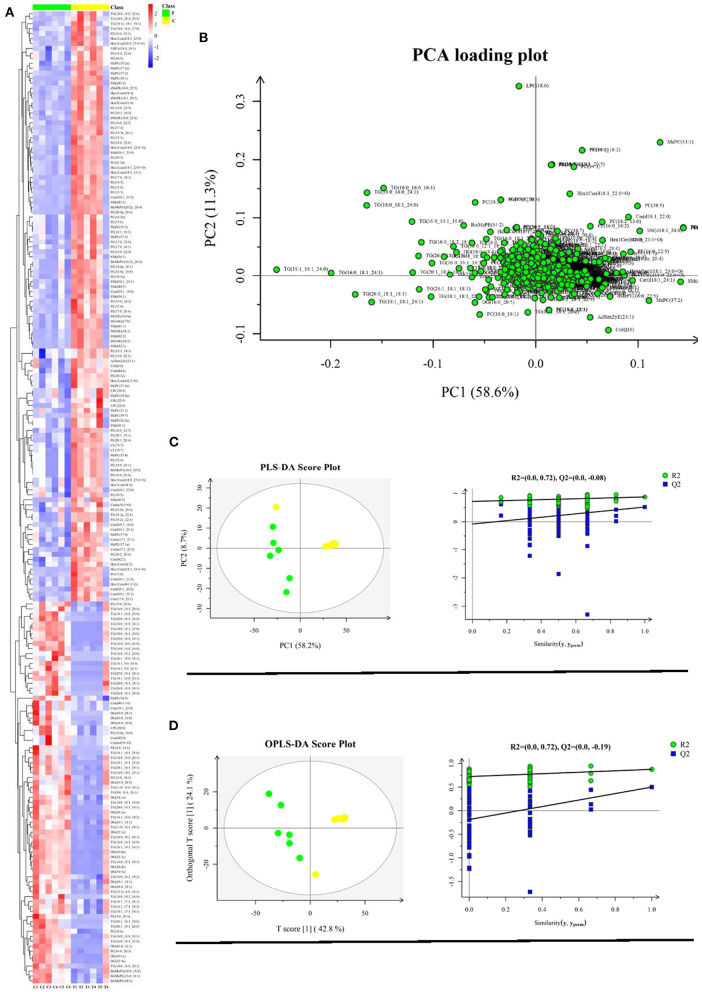
Liver lipidome profile analysis (control group vs. treatment group) (*n* = 6). **(A)** Hierarchical clustering analysis for the significantly different lipid; **(B)** Lipidome profile principal component analysis (PCA); **(C)** PLS-DA score plots and PLS-DA corresponding validation plots; **(D)** OPLS-DA score plots and OPLS-DA corresponding validation plots. Yellow plot represents control group (*n* = 6); green plot represents treatment group (*n* = 6).

The significantly different lipids were screened from the OPLS-DA model (VIP > 1.0 and *p* < 0.05). The different lipids involved in glycerolipids, sphingolipids, glycerophospholipids, and lyso-glycerophospholipids are shown in Supplementary Figures S7–S21. Glycerolipids included triglyceride (TG), diglyceride (DG), monogalactosyldiacylglycerol (MGDG), and monogalactosylmonoacylglycerol (MGMG); sphingolipids included ceramides (Cer), hexaglycosylceramides (Hex1Cer), and sphingomyelins (SM); glycerophospholipids and lyso-glycerophospholipids included phosphatidic acids (PA), phosphatidylinositols (PI), phosphatidylcholines (PC), phosphatidylethanolamines (PE), and LPC. Different lipids amounting to 196 were yielded. Compared with the control group, the treatment group was higher in the levels of TG and DG, and lower in the levels of Hex1Cer, PE, and SM (*P* < 0.05). Compared with the control group, the treatment group was higher in the levels of LPC(20_0), PI(18:0_18:1), and PI(18:0_20:3), while lower in the levels of LPC(20_4), LPC(20_6), and LPC(20_6) (*p* < 0.05). Compared with the control group, the treatment group was higher in the levels of Cer(d40:1+O), Cer(d42:0), Cer(m42:0+O), and Cer(t18:1_22:0), and lower in the levels of Cer(d18:1_21:0), Cer(d40:1+O), Cer(d42:2), Cer(d18:1_25:1), Cer(m34:0+O), Cer(t17:0_25:1), Cer(d18:1_16:0), Cer(d18:1_18:0), Cer(d18:1_22:0), Cer(d18:1_23:3), Cer(d18:1_24:1), Cer(d18:1_24:2), and Cer(d44:6) (*p* < 0.05). Compared with the control group, the treatment group was higher in the levels of PC(16:0_20:3) and PC(18:0_18:1) and lower in the levels of PC(10:0e_10:0), PC(31:0), PC(33:0), PC(33:1), PC(16:0e_18:0), PC(34:2e), PC(35:0), PC(17:0_18:1), PC(35:3), PC(35:4), PC(35:6), PC(36:1e), C(18:1_18:1) PC(19:0_18:2), PC(37:4), PC(15:0_22:6), PC(18:3e_20:1), PC(38:5), PC(16:0_22:6), PC(38:6), PC(39:5), PC(40:3e), PC(18:0_22:4), PC(40:5), PC(18:0_22:6), PC(40:9), PC(41:5e), PC(16:0e_20:4), PC(20:3_18:2), and PC(18:0_22:5) (*p* < 0.05). According to the relative abundance of the significantly different lipids identified, the hierarchical clustering heatmap was generated (Figure 3A). The Z-score plot and the hierarchical clustering heatmap presented similar difference distributions of lipids content (Supplementary Figure S6). The PCA loading plot displayed the lipid profile distribution, which suggested that dietary supplementation with 10% fructose changed the lipid profiles of overfed goose livers (Figure 3B). To better understand the classification and higher level of group separation between two groups, the PLS-DA and OPLS-DA model were used to clarify the different lipidomic patterns. PLS-DA and OPLS-DA score plots showed a clear separation and discrimination between the control group and treatment group (Figures 3C, D). In the PLS-DA model, R2Y and Q2 intercept values were 0.72 and −0.08. The low values of the Q2 intercept represent that the robustness of the model presents a low risk of overfitting and reliability (16). The Q2 values were all <0 in our tests, which indicated that the PLS-DA model could identify the lipidomic pattern differences between the control group and treatment group. The OPLS-DA model further validated the separation and discrimination *via* permutation. In the OPLS-DA score plot, the comparison results of the two groups were more obviously separated.

### Conjoint analysis between transcriptome and lipidome in goose fatty liver

**Figure 4:**
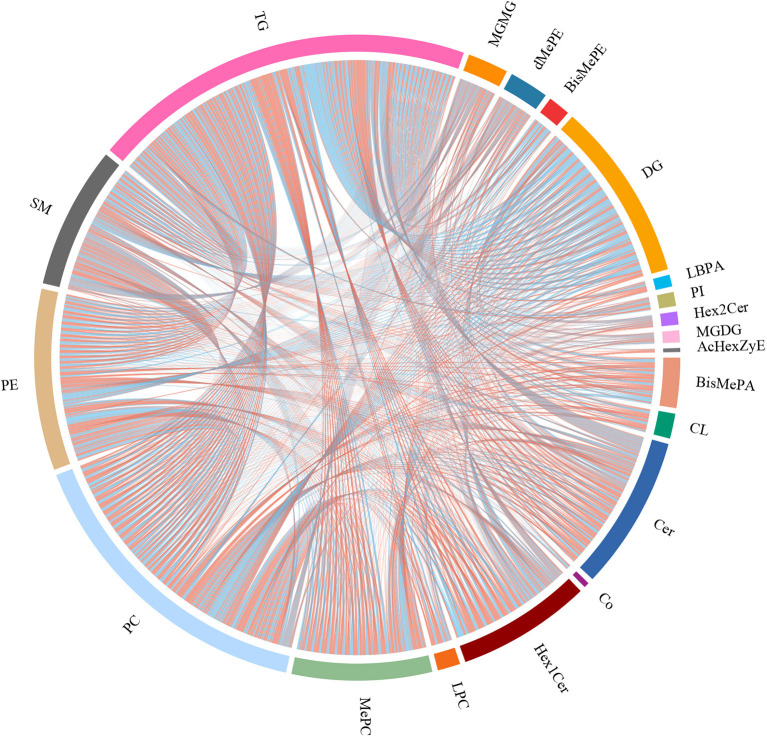
Correlation analysis between different lipids–chordal graph. The line represents the Pearson correlation information of expression values among the lipids, red represents positive correlation, green represents negative correlation, and the darker the color or thicker the line, the higher the correlation intensity.

**Figure 5:**
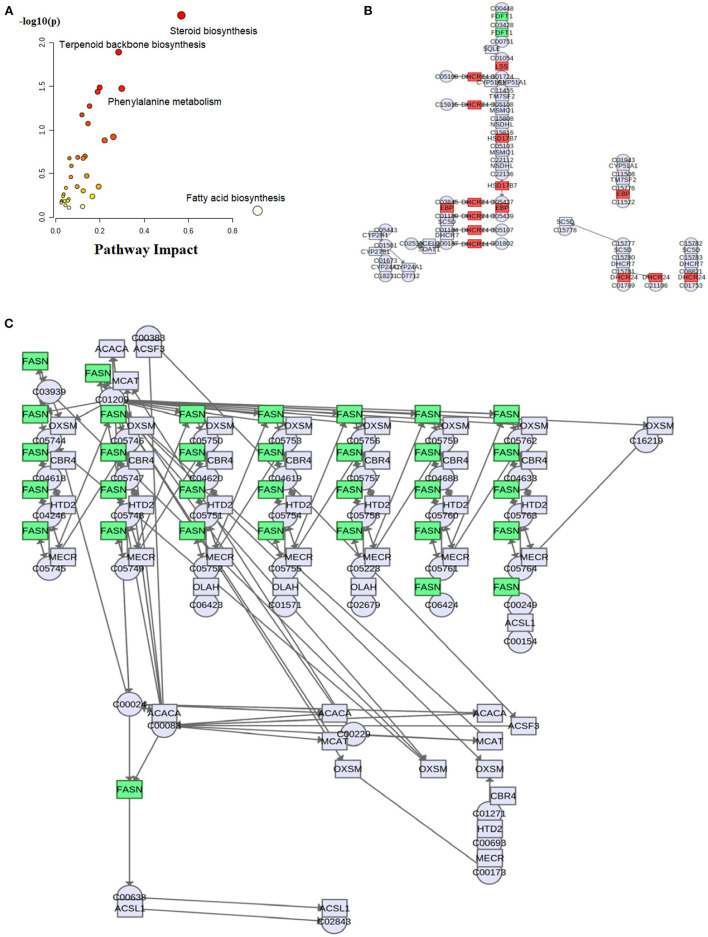
Visualization of conjoint analysis between liver transcriptome and lipidome. **(A)** Enrichment analysis map of significant metabolic pathways; **(B)** steroid biosynthesis pathway; **(C)** fatty acid biosynthesis pathway. These results were generated from MetaboAnalyst 5.0 (https://www.metaboanalyst.ca/) Joint Pathway Analysis.

**Figure 6:**
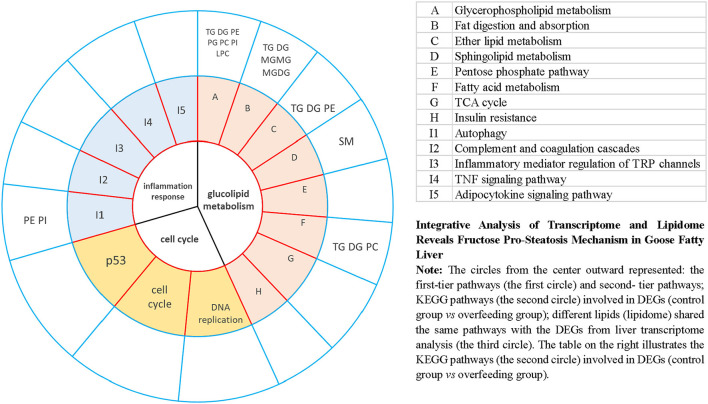
Integrative analysis of transcriptome and lipidome provides insight into goose fatty liver formation *via* KEGG annotation.

To explore the fructose pro-steatosis mechanism from the inter-relationship between different lipids, the correlation analysis between liver different lipids was performed. The correlation analysis between different lipids was presented as a chordal graph (Figure 4) and heatmap (Supplementary Figure S22). The line represented the Pearson correlation information of expression values among the lipids, where red represents positive correlation, green represents negative correlation, and the darker the color or thicker the line represents higher correlation intensity. TG was positively correlated with DG; SM was positively associated with Cer and Hex1Cer. DG was significantly negatively correlated with PC and PE. To further integrate the liver transcriptome and lipidome, MetaboAnalyst5.0 using Joint Pathway Analysis was used to visualize the interrelation between transcriptome and lipidome (Figure 5). Based on the pathway impact scores and -ln *p*-value, the main metabolic pathways were screened and shown in the metabolome view map (Figure 5A and Supplementary Table S4); these pathways were mainly involved in glycerolipid metabolism, glycerophospholipid metabolism, steroid metabolism, glucose metabolism, TCA cycle, fatty acids metabolism (fatty acid elongation and fatty acid biosynthesis), and amino acids metabolism (lysine degradation, glycine, serine, and threonine metabolism and alanine, aspartate and glutamate metabolism, and so forth). Fatty acid biosynthesis and steroid biosynthesis had the highest impact score pathways. The interaction network diagram involved in fatty acid biosynthesis and steroid biosynthesis are shown in Figures 5B, C. Figure 6 integrated the transcriptome analysis and lipidome analysis *via* KEGG annotation. Different lipids mainly mediated these lipid metabolism pathways: glycerophospholipid metabolism, sphingolipid metabolism, and ether lipid metabolism. PC also mediated the metabolism of fatty acids. Notably, PE and PI mediated the autophagy pathway.

## Discussion

TG, monoacylglycerol (MG), and DG belong to the fatty acylglycerol group, which is often referred to as fat. DG is also an important substrate for the biosynthesis of TG, PC, or PE. PC and PE are important components of cell membrane structure. When the produced TG far exceeds the transportation capacity of apolipoproteins, lipids will accumulate in the liver. The imbalance between lipids synthesis and transportation is the main reason for the formation of goose fatty liver (17). Lipidome analysis results demonstrated that dietary supplementation with 10% fructose increased liver TG level, which consisted of the comparison of liver weight, crude fat content, and Oil Red O slice. Sphingolipids are not only components of cell membranes but also bioactivates which participate in physiological processes such as cell proliferation, differentiation, gene expression, and apoptosis. Dietary supplementation with 10% fructose reduced the levels of SM, Cer, and Hex1Cer in overfed geese, which partially reflected the relationship between SM, Cer, and Hex1Cer. Cellular sphingolipid metabolism is centered on ceramide, including ceramide *de novo* synthesis and degradation, and complex sphingolipids synthesis (18). Ceramide is synthesized in the endoplasmic reticulum, and it can be transported to Golgi by the ceramide transporter (CERT) or sphingomyelin and glucosylceramide vesicles which are synthesized *via* sphingomyelin synthase (SMS1) (19). Ceramide is involved in the occurrence and development of NAFLD. Ceramide is one of the inducing factors of insulin resistance and inflammation, and eventually leads to the occurrence and deterioration of non-alcoholic steatohepatitis (NASH) (20). The accumulation of Cer can induce oxidative stress and inflammation in the cell (21). Many research works demonstrated that anti-inflammation is one of the tolerance mechanisms in severe hepatic steatosis during goose fatty liver formation (22, 23). In this study, dietary supplementation with 10% fructose reduced the Cer level, which suggested that fructose greater attenuated the inflammation activated-by Cer and promoted more lipid deposition in overfed geese livers.

Correspondingly, liver transcriptome analysis showed that DEGs involved in inflammatory and immune response albumin (*ALB*) and alanine transaminase (*ALT*) were down regulated. In respect of liver inflammation assessment, *ALB, ALT*, and aspartate transaminase (*AST*), traditional liver enzymes, have been used as clinical evaluation parameters for hepatocyte damage assessment (24, 25). Complement activation is the basic pathological reaction in inflammatory diseases. A previous study demonstrated that the complement system was suppressed by increasing the content of lactic acid in overfed goose liver (23). The anti-inflammation response is considered the protective mechanism which makes geese adaptive to lipid accumulation in the liver (22). In this study, dietary supplementation with 10% fructose upregulated the gene expression levels of *LAG-3* and *CFH* in overfed goose liver. Complement factor H (*CFH* or *BTA*) creates critical negative feedback in alternative pathways which are activated by complement. When *BTA* attaches to complement factor C3b, *BTA* inhibited the formation of membrane attack complex and prevented cell lysis. *BTA* interfered with the complement cascade and protected cells from attacking (26). The production of *BTA* makes the hepatocytes escape from the surveillance of the immune system. In addition, lymphocyte activation gene-3 (*LAG-3*) regulated the immunosuppressive efficacy mediated-by regulatory T cells, which in turn suppressed the immune response (27). Liver lipidome analysis and transcriptome analysis both suggested that dietary supplementation with 10% fructose greater suppressed the inflammatory response and promoted more lipid deposition in overfed goose liver. In addition, dietary supplementation with 10% fructose upregulated the gene expression levels of these key genes involved in cell growth and proliferation in goose liver and induced more lipid deposition in the liver of overfed geese. These results regarding the cell cycle also further addressed the speculation that dietary supplementation with 10% fructose maximized goose liver lipid deposition in advance.

The integrative analysis between transcriptome and lipidome showed that fatty acid biosynthesis and steroid biosynthesis had the highest impact score pathways. The DEGs (*FASN, ELOVL6, SCD*, and *acs*) involved in fatty acid synthesis have all been discussed in connection to overfed geese (28). Under normal circumstances, the synergy of DEGs upregulation will promote more fat accumulation in the liver. However, dietary supplementation with 10% fructose significantly reduced the gene expression levels of key enzymes involved in hepatocyte fatty acids synthesis and steroid biosynthesis (*ELOVL6, SCD, FASN, SREBP1*, and *acs*). This seems to contradict our results that fructose promoted more lipid deposition in the goose liver. Combined with the average overfeeding time, different lipids, and DEGs involved in anti-inflammation response as discussed above, we speculated that dietary supplementation with 10% fructose maximized overfed goose liver lipid deposition in advance. This speculation was consistent with the comparison of overfeeding time and liver color. Dietary supplementation with 10% fructose shortened the average amount of overfeeding time. The liver color of the treatment group was fatty liver color (yellow or khaki), but the liver color of the control group was sandy beige, which suggested that fat accumulation did not reach its maximum in the lives of geese only overfed with maize flour. The lipid deposition process in the lives of geese only overfed with maize flour would continue until it reached its maximum, and the expression levels of genes involved in liver lipid synthesis would be upregulated. However, after fat accumulation was saturated, the expression levels of genes involved in hepatocytes lipid synthesis were downregulated and the lipid accumulation slowed down or stopped in the livers of geese overfed with maize flour supplemented with 10% fructose. Therefore, the key DEGs involved in fatty acid synthesis and steroid biosynthesis were downregulated. In addition, the risk for inflammation also increased after the fat accumulation levels reached to maxization in the goose liver. Previous research results evidenced that waterfowl had evolved a series of mechanisms to protect their liver from severe hepatic steatosis in the process of adaptation (29). When the harm caused by severe hepatic steatosis increases, the protective mechanism will be activated. As discussed above, dietary supplementation with 10% fructose decreased the Cer level, upregulated the gene expression levels of DEGs involved in the anti-inflammatory response, and downregulated gene expression levels of DEGs involved in the proinflammatory response in the liver. Therefore, liver inflammatory response presented greater inhibition in the geese of the treatment group from transcriptome analysis and lipidome analysis.

It is well-known that the liver is the primary position of lipid biosynthesis in birds, and lipid synthesis concentrates in the liver for the overfed goose. The lipids synthesized in the liver are mostly transported by very low-density lipoproteins (*VLDL*), and the lipids in the diet are primarily transported by chylomicrons. After being hydrolyzed by lipoprotein lipase (*LPL*) in the blood, most of the chylomicrons and lipids transported by *VLDL* are deposited in peripheral adipose tissues and muscles (28). As discussed above, fructose stronger promotes lipid deposition in goose fatty liver formation. When the lipid deposition was saturated in the liver of an overfed goose, excessive lipids converted from received carbohydrates during overfeeding will be transported to peripheral adipose tissue. Dietary supplementation with 10% fructose upregulated *LPL* expression levels in liver and intestine-mesentery fat tissue and then induced more lipid transportation and deposition in peripheral adipose tissue. Peripheral adipose tissue can be treated as a constituent part of the protective mechanism which prevents the development from simple hepatic steatosis to more advanced hepatic pathological changes. A study involving pigs reported that high-fructose feeding upregulated hepatic *de novo* lipogenesis enzymes (*ACACA* and *FASN*), and pigs utilized adipose tissue as the main *de novo* lipogenesis organ (30). In the current study, dietary supplementation with 10% fructose upregulated the expression levels of key genes involved in lipid biosynthesis (*G6PC, glpK*, dgkA, and *ACSL*) in peripheral adipose tissues and promoted more lipids synthesis, which suggested that overfed geese were protected against steatosis induced by fructose by depending on peripheral adipose tissues for *de novo* lipogenesis. On the other hand, dietary supplementation with 10% fructose upregulated the gene expression levels of these key genes involved in lipid synthesis and transportation in goose liver and peripheral adipose tissues and promoted more lipids deposited in peripheral adipose tissues in the relatively short overfeeding period. There was no significant difference between the control group and the treatment group in peripheral adipose tissue weight. These results also further illustrated our speculation that dietary supplementation with 10% fructose maximized goose liver lipid deposition in advance.

## Conclusion

In summary, this study revealed the fructose pro-steatosis mechanism from transcriptome and lipidome in goose fatty liver formation. The conjoint analysis between transcriptome and lipidome showed that fatty acid biosynthesis and steroid biosynthesis were the highest impact score pathways. Dietary supplementation with 10% fructose greatly increased lipid synthesis and cell growth and proliferation, and suppressed the inflammatory response and induced more lipid deposition in the liver. Lipid accumulation reaches maximum levels in the lives of geese overfed with maize flour supplemented with 10% fructose sooner than geese only overfed with maize flour. However, further research is needed to verify this speculation, for example, investigating the alteration of liver lipidome and transcriptome at different points during the overfeeding period. Nevertheless, the method of dietary supplementation with 10% fructose (overfeeding five times per day) can effectively promote lipid accumulation in overfed geese and shorten the overfeeding time. This method not only improves the production efficiency and quality of *foie gras* but also will be conducive to animal welfare in *foie gras* production.

## Data availability statement

The original contributions presented in the study are publicly available. This data can be found here: https://figshare.com/articles/dataset/Integrative_Analysis_between_Transcriptome_and_Lipidome_Reveal_Fructose_Pro-Steatosis_Mechanism_in_Goose_Fatty_Liver_Formation/21060628/1.

## Ethics statement

The animal study was reviewed and approved by Institutional Animal Care and Use Committee (IACUC) of Sichuan Agricultural University (Permit No. DKY-B20141401). Written informed consent was obtained from the owners for the participation of their animals in this study.

## Author contributions

RW: methodology, investigation, data curation, writing the original draft, and formal analysis. CH: conceptualization, methodology, resources, supervision, project administration, funding acquisition, and writing—review and editing. SW and LL: methodology, investigation, and data curation. YT, SH, and HL: investigation. BK: resources. HX: funding acquisition. All authors contributed to the article and approved the submitted version.

## References

[1] Lee J Oh AR Lee HY Moon YA Lee HJ Cha JY. Deletion of KLF10 leads to stress-induced liver fibrosis upon high sucrose feeding. Int J Mol Sci. (2021) 22:331. 10.3390/ijms22010331 33396939 PMC7794950

[2] Wang YL Zhou X Li DL Ye JM. Role of the mtor-autophagy-ER stress pathway in high fructose-induced metabolic-associated fatty liver disease. Acta Pharmacol Sin. (2022) 43:10–4. 10.1038/s41401-021-00629-0 33731774 PMC8724298

[3] Geng T Zhao X Xia L Liu L Li F Yang B. Supplementing dietary sugar promotes endoplasmic reticulum stress-independent insulin resistance and fatty liver in goose. Biochem Biophys Res Commun. (2016) 476:665–9. 10.1016/j.bbrc.2016.05.149 27246737

[4] Yu X Ren LP Wang C Zhu YJ Xing HY Zhao J. Role of X-box binding protein- in fructose-induced de novo lipogenesis in HepG2 cells. Chin Med J. (2018) 131:2310–9. 10.4103/0366-6999.241799 30246717 PMC6166463

[5] Montgomery MK Fiveash CE Braude JP Osborne B Brown SHJ Mitchell TW. Disparate metabolic response to fructose feeding between different mouse strains. Sci Rep. (2015) 5:18474. 10.1038/srep18474 26690387 PMC4686880

[6] Miura K Ohnishi H. Role of gut microbiota and toll-like receptors in nonalcoholic fatty liver disease. World J Gastroenterol. (2014) 20:7381–91. 10.3748/wjg.v20.i23.7381 24966608 PMC4064083

[7] Jang C Wada S Yang S Gosis B Zeng XF Zhang ZY. The small intestine shields the liver from fructose-induced steatosis. Nat Metabol. (2020) 2:586–93. 10.1038/s42255-020-0222-9 32694791 PMC8020332

[8] Todoric J Di Caro G Reibe S Henstridge DC Green CR Vrbanac A. Fructose stimulated de novo lipogenesis is promoted by inflammation. Nat Metabol. (2020) 2:1034–45. 10.1038/s42255-020-0261-2 32839596 PMC8018782

[9] Hermier D Salichon MR Guy G Peresson R. Differential channelling of liver lipids in relation to susceptibility to hepatic steatosis in the goose. Poult Sci. (1999) 78:1398–406. 10.1093/ps/78.10.1398 10536788

[10] Xu L Duanmu Y Blake GM Zhang CX Zhang Y Brown K. Validation of goose liver fat measurement by QCT And CSE-MRI with biochemical extraction and pathology ss reference. Eur Radiol. (2018) 28:2003–12. 10.1007/s00330-017-5189-x 29238866

[11] Wei R Deng D Teng Y Lu C Luo Z Abdulai M. Study on the effect of different types of sugar on lipid deposition in goose fatty liver. Poult Sci. (2022) 101:101729. 10.1016/j.psj.2022.101729 35172237 PMC8850742

[12] Lu CC Wei RX Deng DH Luo ZY Abdulai M Liu HH. Effect of different types of sugar on gut physiology and microbiota in overfed goose. Poult Sci. (2021) 100:101208. 10.1016/j.psj.2021.101208 34102480 PMC8187246

[13] Vvedenskaya O Rose TD Knittelfelder O Palladini A. Wodke JaH, Schuhmann K, et al. Nonalcoholic fatty liver disease stratification by liver lipidomics. J Lipid Res. (2021) 62:100104. 10.1016/j.jlr.2021.100104 34384788 PMC8488246

[14] Merli M Lattanzi B Aprile F. Sarcopenic obesity in fatty liver. Curr Opin Clin Nutr Metab Care. (2019) 22:185–90. 10.1097/MCO.0000000000000558 30893090

[15] Mendez-Sanchez N Cesar Cruz-Ramon V Lenin Ramirez-Perez O Hwang JP Barranco-Fragoso B Cordova-Gallardo J. New aspects of lipotoxicity in nonalcoholic steatohepatitis. Int J Mol Sci. (2018) 19:2034. 10.3390/ijms19072034 30011790 PMC6073816

[16] Yu Y Lyu W Fu Z Fan Q Xiao Y Ren Y. Metabolic profiling analysis of liver in landes geese during the formation of fatty liver via GC-TOF/MS. Front Physiol. (2022) 11:581699. 10.3389/fphys.2021.783498 35046836 PMC8761942

[17] Wei R Han C Deng D Ye F Gan X Liu H. Research progress into the physiological changes in metabolic pathways in waterfowl with hepatic steatosis. Br Poult Sci. (2020) 62:118–24. 10.1080/00071668.2020.1812527 32902307

[18] Ghandour B Dbaibo G Darwiche N. The unfolding role of ceramide in coordinating retinoid-based cancer therapy. Biochemical J. (2021) 478:3621–42. 10.1042/BCJ20210368 34648006

[19] Hanada K Kumagai K Tomishige N Yamaji T. CERT-mediated trafficking of ceramide. Biochimica Et Biophysica Acta-Molecular and Cell Biology of Lipids. (2009) 1791:684–91. 10.1016/j.bbalip.2009.01.006 19416656

[20] Regnier M Polizzi A Guillou H Loiseau N. Sphingolipid metabolism in non-alcoholic fatty liver diseases. Biochimie. (2019) 159:9–22. 10.1016/j.biochi.2018.07.021 30071259

[21] Shabbir MA Mehak F Khan ZM Ahmad W Khan MR Zia S. Interplay between Ceramides and Phytonutrients: New Insights in Metabolic Syndrome. Trends Food Sci Technol. (2021) 111:483–94. 10.1016/j.tifs.2021.03.010

[22] Liu L Zhao X Wang Q Sun XX Xia LL Wang QQ. Prosteatotic and protective components in a unique model of fatty liver: gut microbiota and suppressed complement system. Sci Reports. (2016) 6:31763. 10.1038/srep31763 27550859 PMC4994046

[23] Geng T Yang B Li F Xia L Wang Q Zhao X. Identification of protective components that prevent the exacerbation of goose fatty liver: characterization, expression and regulation of adiponectin receptors. Comp Biochem Physiol B. (2016) 194–5:32–38. 10.1016/j.cbpb.2016.01.006 26804769

[24] Durgappa M Saraswat V Pande G Mohindra S Bhadauria AS Butala SP. Slow continuous infusion of diuretic, albumin and/or terlipressin in patients with severe alcoholic hepatitis and acute on chronic liver failure. J Gastroenterol Hepatol. (2019) 34:424–424. 10.1016/j.jceh.2018.06.284

[25] Al-Megrin WA Alkhuriji AF Yousef AOS Metwally DM Habotta OA Kassab RB. Antagonistic efficacy of luteolin against lead acetate exposure-associated with hepatotoxicity is mediated via antioxidant, anti-inflammatory, and anti-apoptotic activities. Antioxidants. (2020) 9:10. 10.3390/antiox9010010 31877779 PMC7022878

[26] Morgan HP Schmidt CQ Guariento M Blaum BS Gillespie D Herbert AP. Structural basis for engagement by complement factor H of C3b on a self surface. Nat Struct Mol Biol. (2011) 18:463–70. 10.1038/nsmb.2018 21317894 PMC3512577

[27] Wang J Sanmamed MF Datar I Su TT Ji L Sun J. Fibrinogen-like protein 1 is a major immune inhibitory ligand of LAG-3. Cell. (2019) 176:334–47. 10.1016/j.cell.2018.11.010 30580966 PMC6365968

[28] Lu L Chen Y Wang Z Li X Chen W Tao Z. The goose genome sequence leads to insights into the evolution of waterfowl and susceptibility to fatty liver. Genome Biol. (2015) 16:89. 10.1186/s13059-015-0652-y 25943208 PMC4419397

[29] Gu W Wen K Yan C Li S Liu T Xu C. Maintaining intestinal structural integrity is a potential protective mechanism against inflammation in goose fatty liver. Poult Sci. (2020) 99:5297–307. 10.1016/j.psj.2020.08.052 33142445 PMC7647926

[30] Schmidt NH Svendsen P Albarran-Juarez J Moestrup SK Bentzon JF. High-fructose feeding does not induce steatosis or non-alcoholic fatty liver disease in pigs. Sci Rep. (2021) 11:2807. 10.1038/s41598-021-82208-1 33531575 PMC7854584

