# Supplementary material for: Integrative analysis of transcriptome and lipidome reveals fructose pro-steatosis mechanism in goose fatty liver
Source: Front Nutr. 2023 Jan 10;9:1052600. doi: 10.3389/fnut.2022.1052600 (PMC9871465; doi:10.3389/fnut.2022.1052600)
Supplement: Supplementary file 1 [file Data_Sheet_1.PDF]

Table S1 Diet formula for experiment

Items	Control group	Treatment group
Feed intake (g / day)	1800-2000	1800-2000
Maize flour（%）	94.5	84.5
Fructose (%)	0	10
Fish Flour（%）	2	2
NaCl（%）	1	1
Soya Oil（%）	2.5	2.5
Total（%）	100	100
Nutrient levels		
ME/（MJ/kg）	13.6	13.6
Crude Protein（%）	8	8
Lysine（%）	0.84	0.85
Methionine（%）	0.41	0.41
Calcium（%）	0.7	0.7
Total Phosphorus（%）	0.54	0.54

Table S2 Sequencing data statistics

	Sample	Reads No.	Bases (bp)	Q30 (bp)	N (%)	Q20 (%)	Q30 (%)
Liver	C1	53262160	7989324000	7559016868	0.000769	97.96	94.61
	C2	54945118	8241767700	7774811689	0.000785	97.77	94.33
	C3	51828122	7774218300	7357044610	0.000781	97.95	94.63
	T1	45315660	6797349000	6392453967	0.000742	97.66	94.04
	T2	46831250	7024687500	6626034913	0.00074	97.81	94.32
	T3	49945898	7491884700	7021173577	0.000725	97.42	93.71
AF	C1	44344194	6651629100	6173539464	0.000227	97.14	92.81
	C2	50947256	7642088400	7120266808	0.000226	97.35	93.17
	C3	48514560	7277184000	6812508876	0.000226	97.53	93.61
	T1	54942958	8241443700	7689118391	0.000229	97.41	93.29
	T2	44883762	6732564300	6257954185	0.000237	97.22	92.95
	T3	50167510	7525126500	7020955533	0.000229	97.39	93.3
IF	C1	55547680	8332152000	7644573299	0.000729	96.42	91.74
	C2	51466002	7719900300	7184857188	0.000727	97.03	93.06
	C3	50189088	7528363200	7060453153	0.000713	97.42	93.78
	T1	53256756	7988513400	7515822944	0.000795	97.7	94.08
	T2	50073176	7510976400	7048436944	0.000792	97.52	93.84
	T3	48182344	7227351600	6794607792	0.000754	97.6	94.01
SF	C1	47662992	7149448800	6677547300	0.000229	97.35	93.39
	C2	48794740	7319211000	6666689480	0.000218	96.29	91.08
	C3	49222716	7383407400	6850446336	0.000228	97.16	92.78
	T1	56680852	8502127800	7921152732	0.000227	97.22	93.16
	T2	51247052	7687057800	7129209453	0.000227	97.03	92.74
	T3	54999522	8249928300	7620533349	0.000226	96.93	92.37

Note: Reads No.: Total number of reads.

Bases(bp): Total number of bases.

Q30 (bp): The total number of bases with a base identification accuracy of 99.9% or more.

N (%): Fuzzy base percentage.

Q20(%): Percentage of bases with a base identification accuracy of 99.9% or more.

Q30(%): Percentage of bases with a base identification accuracy of 99.9% or more.

C = Control group; O = Overfeeding group. SF = subcutaneous fat tissue. AF = abdominal fat tissue. IF = intestine-mesentery fat tissue.

Table S3 Filtering data

	Sample	Clean Reads No.	Clean Data (bp)	Clean Reads %	Clean Data %
Liver	C1	49950414	7492562100	93.78	93.78
	C2	51317526	7697628900	93.39	93.39
	C3	48272974	7240946100	93.14	93.14
	T1	42481704	6372255600	93.74	93.74
	T2	43913536	6587030400	93.76	93.76
	T3	46821252	7023187800	93.74	93.74
AF	C1	41285568	6192835200	93.1	93.1
	C2	46666678	7000001700	91.59	91.59
	C3	45200642	6780096300	93.16	93.16
	T1	50975784	7646367600	92.77	92.77
	T2	41880154	6282023100	93.3	93.3
	T3	47012510	7051876500	93.71	93.71
IF	C1	51315060	7697259000	92.38	92.38
	C2	47806384	7170957600	92.88	92.88
	C3	46592110	6988816500	92.83	92.83
	T1	49445844	7416876600	92.84	92.84
	T2	46684882	7002732300	93.23	93.23
	T3	45081230	6762184500	93.56	93.56
SF	C1	43804752	6570712800	91.9	91.9
	C2	45060596	6759089400	92.34	92.34
	C3	45873308	6880996200	93.19	93.19
	T1	52496976	7874546400	92.61	92.61
	T2	47530004	7129500600	92.74	92.74
	T3	51397212	7709581800	93.45	93.45

Note: Clean Reads No: The number of high-quality reads.

Clean Data (bp): The number of high-quality bases.

Clean Reads %: Percentage of high-quality reads to sequenced reads.

Clean Data %: Percentage of high-quality sequence bases to sequenced bases.

C = Control group; O = Overfeeding group. SF = subcutaneous fat tissue. AF = abdominal fat tissue. IF = intestine-mesentery fat tissue.

Table S4 DEGs involved in glucolipid metabolism and immune response

Liver				AF		IF		SF	
Gene name	up/ down	Gene name	up/ down	Gene name	up/ down	Gene name	up/ down	Gene name	up/ down
PDL1	up	CASP3	down	PERP	up	ACSL	up	G6PC	up
LPL	up	pckA	down	LDLR	up	CD44	up	dgkA	up
CASP1	up	FASN	down	CH25H	up	CD36	up	CCNA	up
MAPT	up	ELOVL6	down	LMNB	up	lip	up	glpK	up
ALDH1L	up	SULT2B	down	glmS	up	glpK	up	ACSL	up
HES1	up	ALT	down	ACSL	up	LPL	up	CDC45	up
AOC3	up	ALB	down	ALDH3	up	CD99	up	TN	up
MICAL	up	DAPK	down	E2.3.3.10	up	LRP1	up	PYG	up
B4GALNT4	up	CTSE	down	FABP7	up	dgkA	up	PDK2	down
TRA1	up	acs	down	FOXO3	up	CDK5	down	AACS	down
IL9	up	FOXP3	down	LEPR	up	DAXX	down	SREBP1	down
CYP2K	up	PERP	down	gltA	up	glpK	down	GPD1	down
AOC3	up	PAK3	down	CCRK	up	GAPDH	down	SRD5A2	down
CMPK2	up	SCD	down	GPD1	up	GCGR	down	AKR1A1	down
PRF1	up	AMPK	down	SCD	down	pgk	down	HMGCR	down
G6PC	up	HMGCR	down	FABP4	down	CYP51	down		
IL20RB	up	FADS2	down	HGF	down	GPR20	down		
IDH1	up	N6AMT1	down	GTF2I	down	GPD1	down		
P21	up	fabF	down	LPCAT3	down				
RASA1	up	SREBP1	down	ACK1	down				
KRAB	up	HSD17B7	down	CD82	down				
IL36RN	up	MESO1	down	glgP	down				
NFKBID	up	TP53I3	down	SOAT	down				
CMPK2	up			AGPAT6	down				
CFH	up			GLUT4	down				
DDHD1	up			AASDH	down				
dgkA	up			ALDH3	down				
PLD1_2	up								
DGAT2	up								
ACS	up								
LAG3	up								

Table S4 Metabolic pathways identified on integrative analysis between transcriptome and lipidome

	Total	Expected	Hits	Raw p	- log p	Holm adjus	FDR	Impact
Fatty acid biosynthesis	129	1.80	1	0.84	0.074	1	1	0.914
Steroid biosynthesis	82	1.14	5	0.00	2.310	0.4117	0.41	0.568
Phenylalanine metabolism	21	0.29	2	0.03	1.474	1	0.61	0.300
Terpenoid backbone biosynthesis	36	0.50	3	0.01	1.889	1	0.54	0.286
Starch and sucrose metabolism	43	0.60	2	0.12	0.923	1	1	0.262
Nitrogen metabolism	10	0.14	1	0.13	0.882	1	1	0.222
Galactose metabolism	51	0.71	3	0.03	1.486	1	0.61	0.200
Citrate cycle (TCA cycle)	42	0.59	1	0.45	0.349	1	1	0.195
alpha-Linolenic acid metabolism	22	0.31	2	0.04	1.437	1	0.61	0.190
Folate biosynthesis	61	0.85	1	0.58	0.237	1	1	0.167
Arginine biosynthesis	27	0.38	2	0.05	1.274	1	0.75	0.154
Glycerolipid metabolism	35	0.49	2	0.08	1.074	1	0.88	0.147
Glycerophospholipid metabolism	86	1.20	2	0.34	0.470	1	1	0.141
Taurine and hypotaurine metabolism	16	0.22	1	0.20	0.695	1	1	0.133
Ubiquinone and other terpenoid-quinone biosynthesis	17	0.24	1	0.21	0.672	1	1	0.125
Lysine degradation	49	0.68	1	0.50	0.300	1	1	0.125
Pyrimidine metabolism	99	1.38	1	0.76	0.120	1	1	0.122
Glycine, serine and threonine metabolism	68	0.95	3	0.07	1.174	1	0.80	0.119
Alanine, aspartate and glutamate metabolism	61	0.85	2	0.21	0.682	1	1	0.100
Nicotinate and nicotinamide metabolism	42	0.59	1	0.45	0.349	1	1	0.098
Cysteine and methionine metabolism	71	0.99	2	0.26	0.585	1	1	0.071
Tyrosine metabolism	88	1.23	2	0.35	0.457	1	1	0.069
Linoleic acid metabolism	17	0.24	1	0.21	0.672	1	1	0.063
Steroid hormone biosynthesis	199	2.77	2	0.78	0.108	1	1	0.061
Inositol phosphate metabolism	69	0.96	1	0.63	0.204	1	1	0.059
Phosphatidylinositol signaling system	74	1.03	1	0.65	0.186	1	1	0.055
beta-Alanine metabolism	44	0.61	1	0.46	0.334	1	1	0.047
Primary bile acid biosynthesis	92	1.28	1	0.73	0.136	1	1	0.044
Fatty acid elongation	75	1.05	1	0.66	0.183	1	1	0.041
Glutathione metabolism	56	0.78	1	0.55	0.261	1	1	0.036
Glyoxylate and dicarboxylate metabolism	56	0.78	1	0.55	0.261	1	1	0.036
Glycolysis or Gluconeogenesis	61	0.85	1	0.58	0.237	1	1	0.033
Aminoacyl-tRNA biosynthesis	74	1.03	1	0.65	0.186	1	1	0.027
Arginine and proline metabolism	78	1.09	1	0.67	0.173	1	1	0.026
Arachidonic acid metabolism	81	1.13	1	0.69	0.164	1	1	0.025
