# Supplementary material for: Integrative analysis of transcriptome and lipidome reveals fructose pro-steatosis mechanism in goose fatty liver
Source: Front Nutr. 2023 Jan 10;9:1052600. doi: 10.3389/fnut.2022.1052600 (PMC9871465; doi:10.3389/fnut.2022.1052600)
Supplement: Supplementary file 2 [file Data_Sheet_2.PDF]

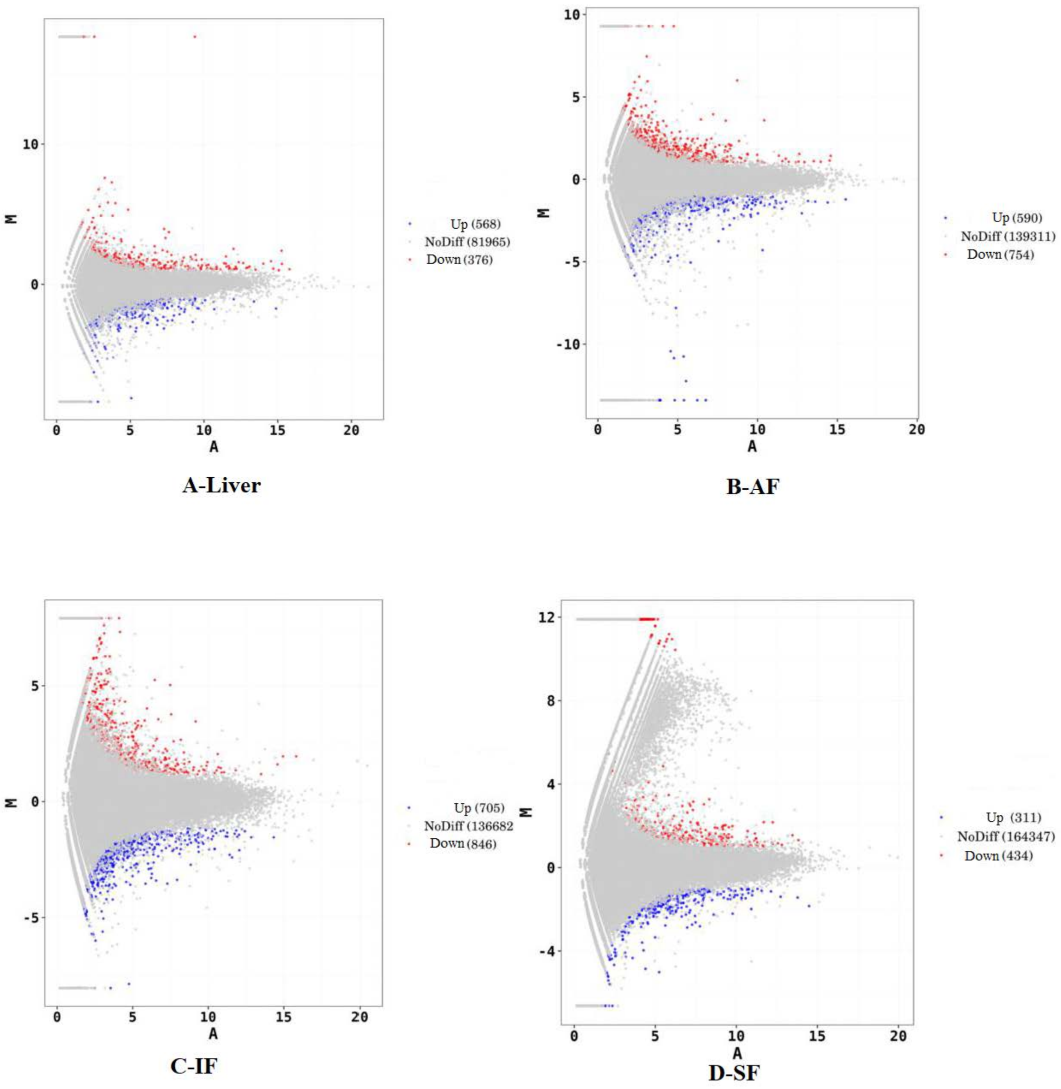

Figure S1 MA plots of DEGS came from transcriptome analysis (control *vs* treatment) (n=3). A, MA plots of DEGS in liver tissue. B, MA plots of DEGS in abdomen fat tissue; C, MA plots of DEGS in intestine-mesentery fat tissue. D, MA plots of DEGS in subcutaneous fat tissue;

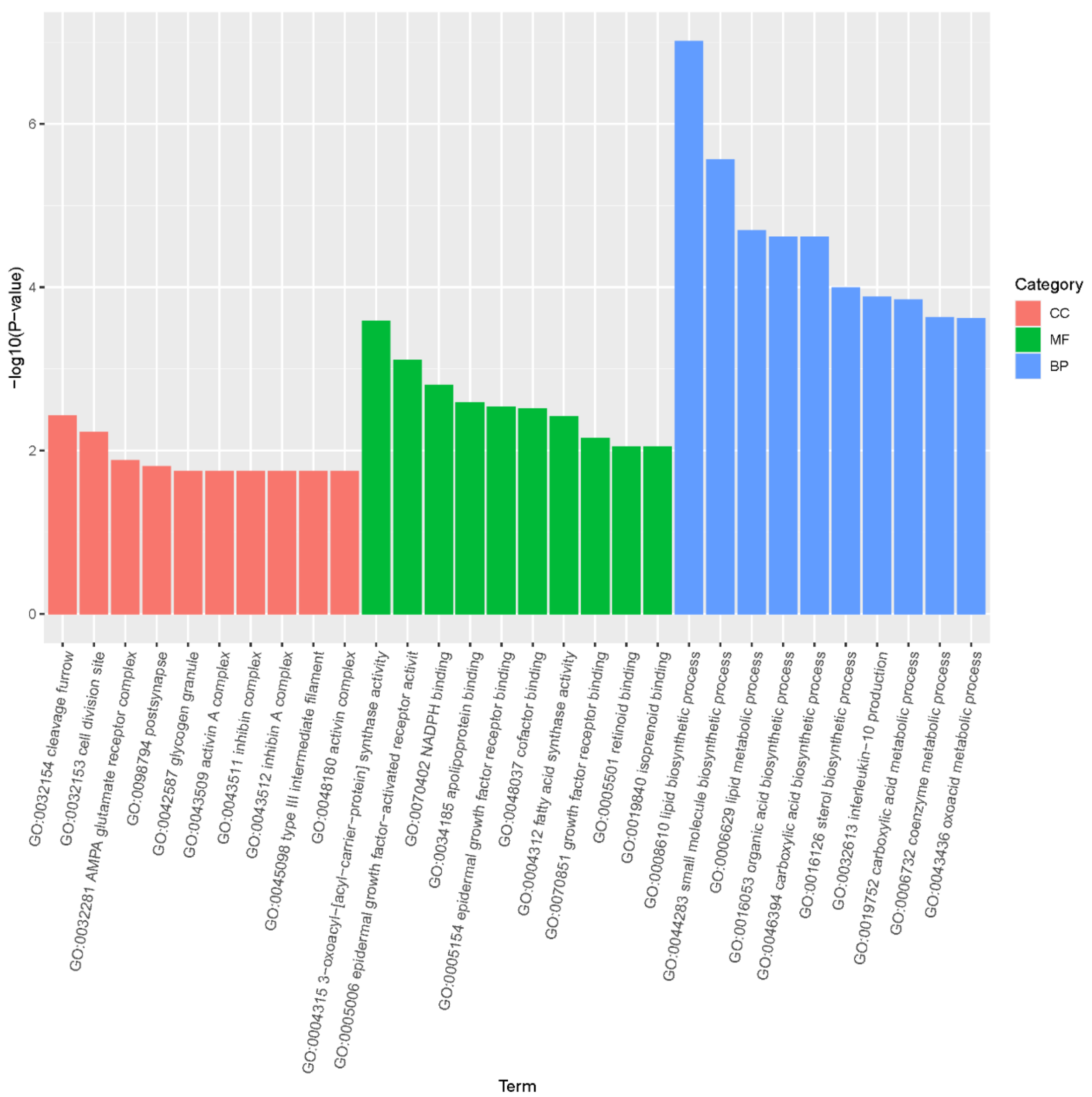

Figure S2 GO analysis of liver tissue

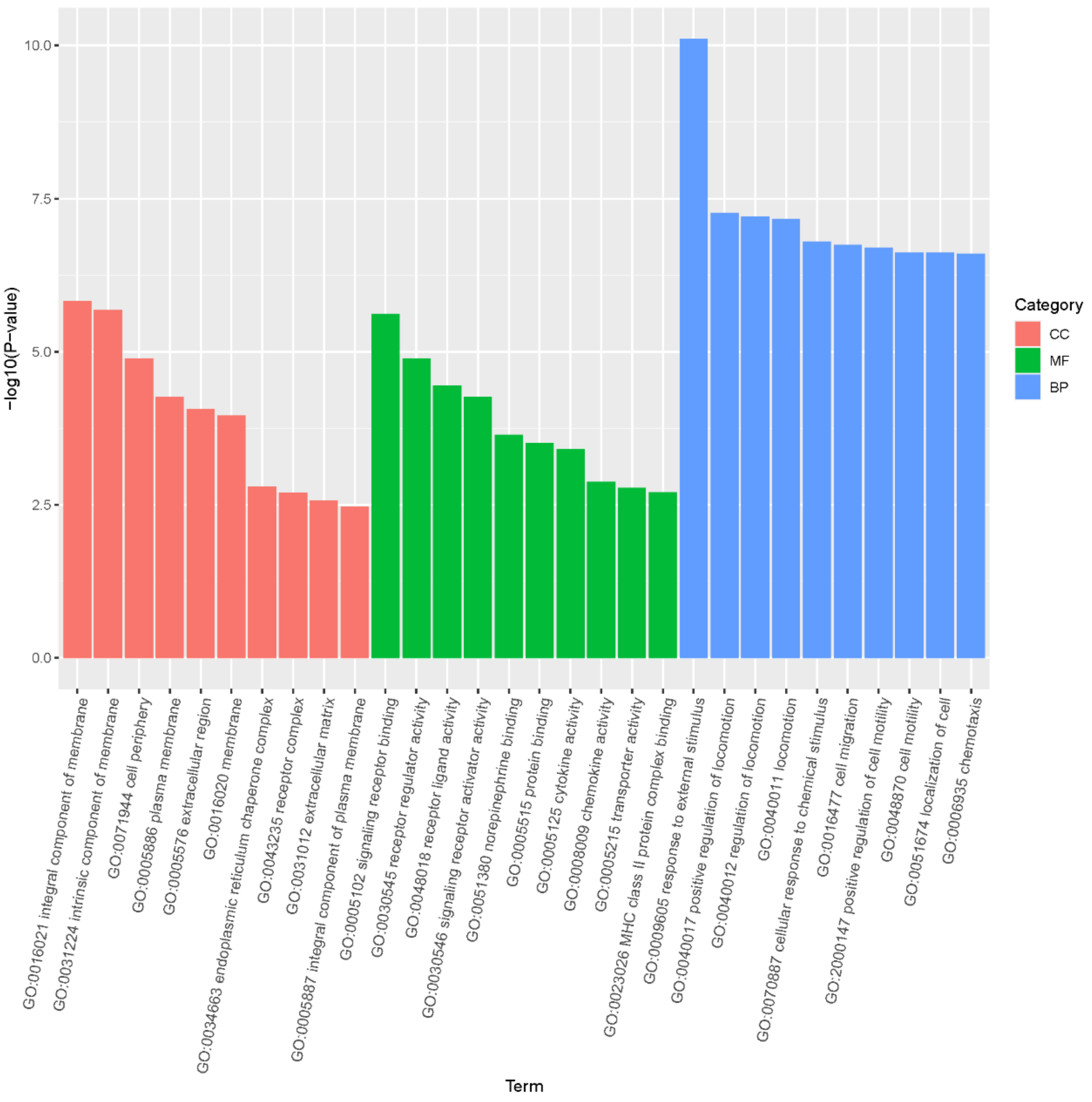

Figure S3 GO analysis of abdomen fat tissue

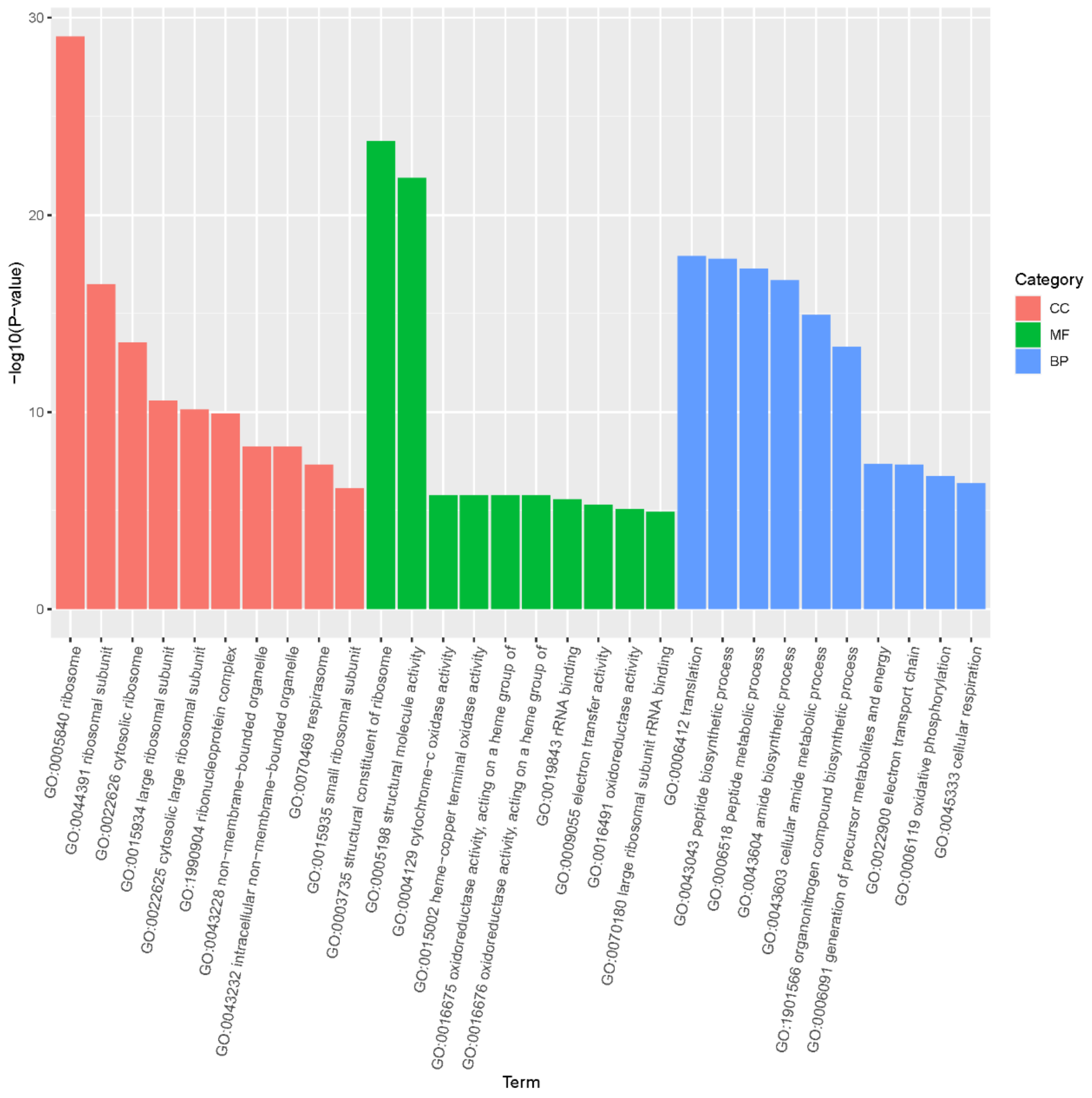

Figure S4 GO analysis of intestine-mesentery tissue

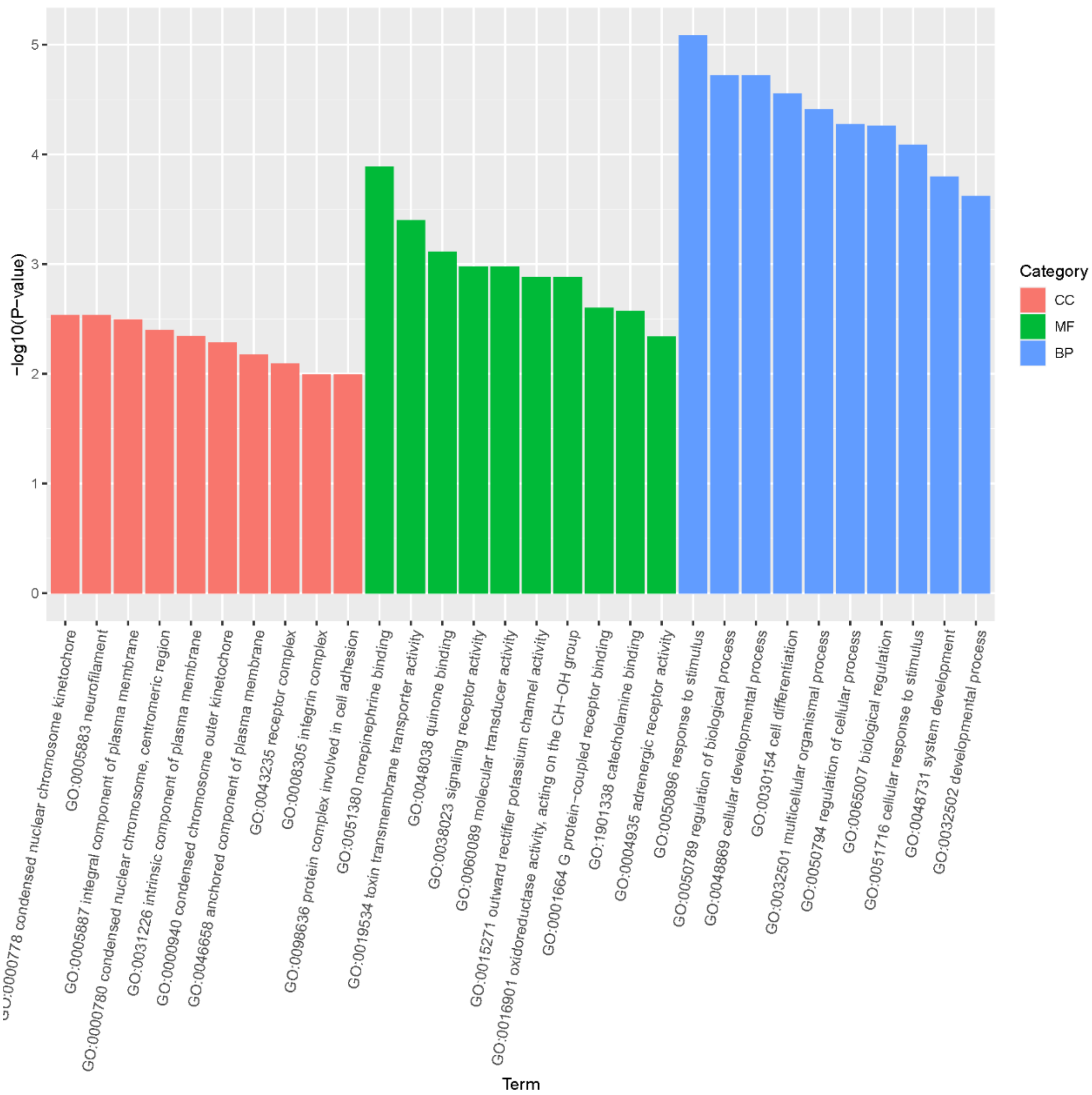

Figure S5 GO analysis of subcutaneous fat tissue

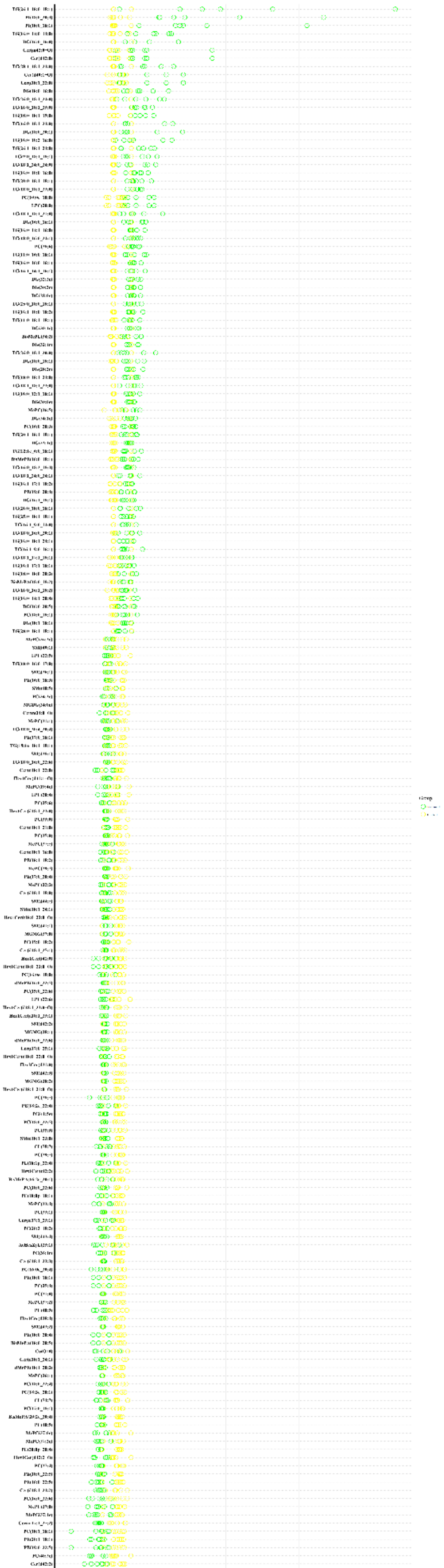

Figure S6 Liver lipidome Z score

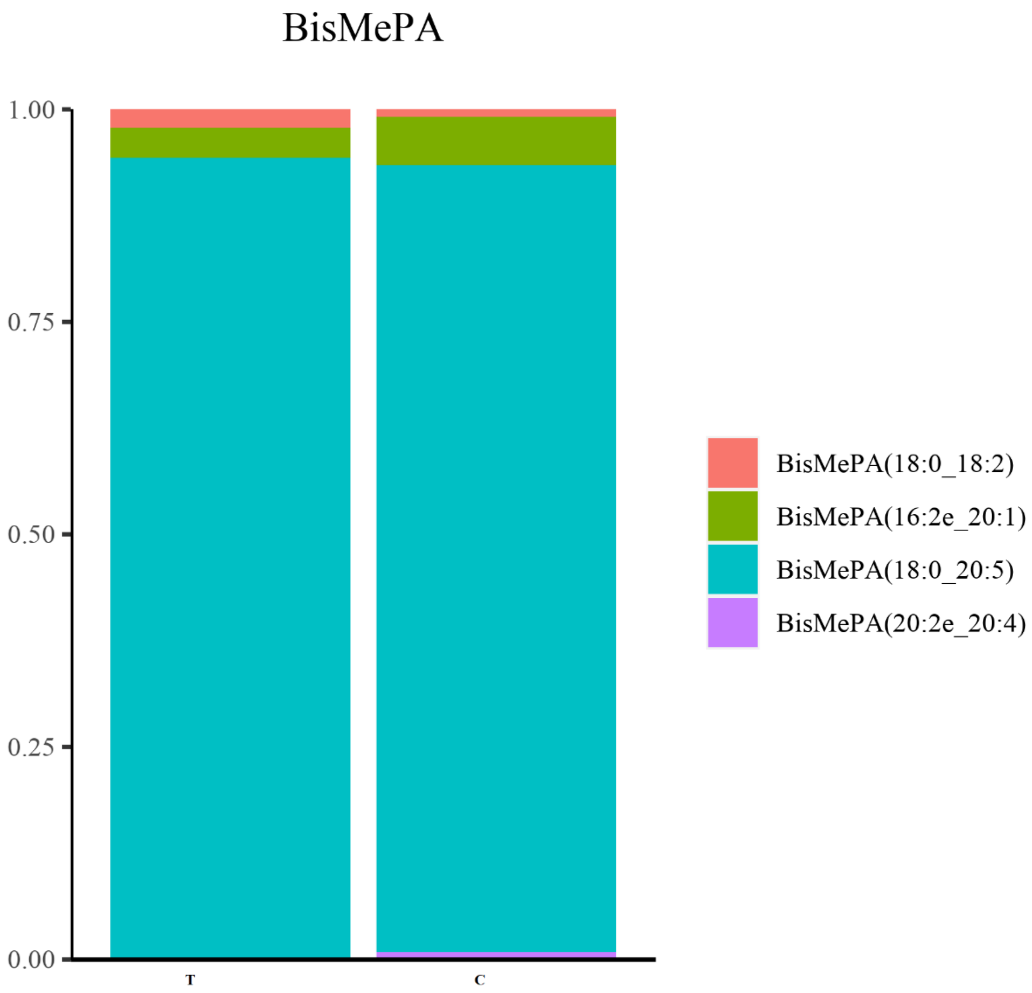

Figure S7 Liver lipidome different lipids- BisMePA

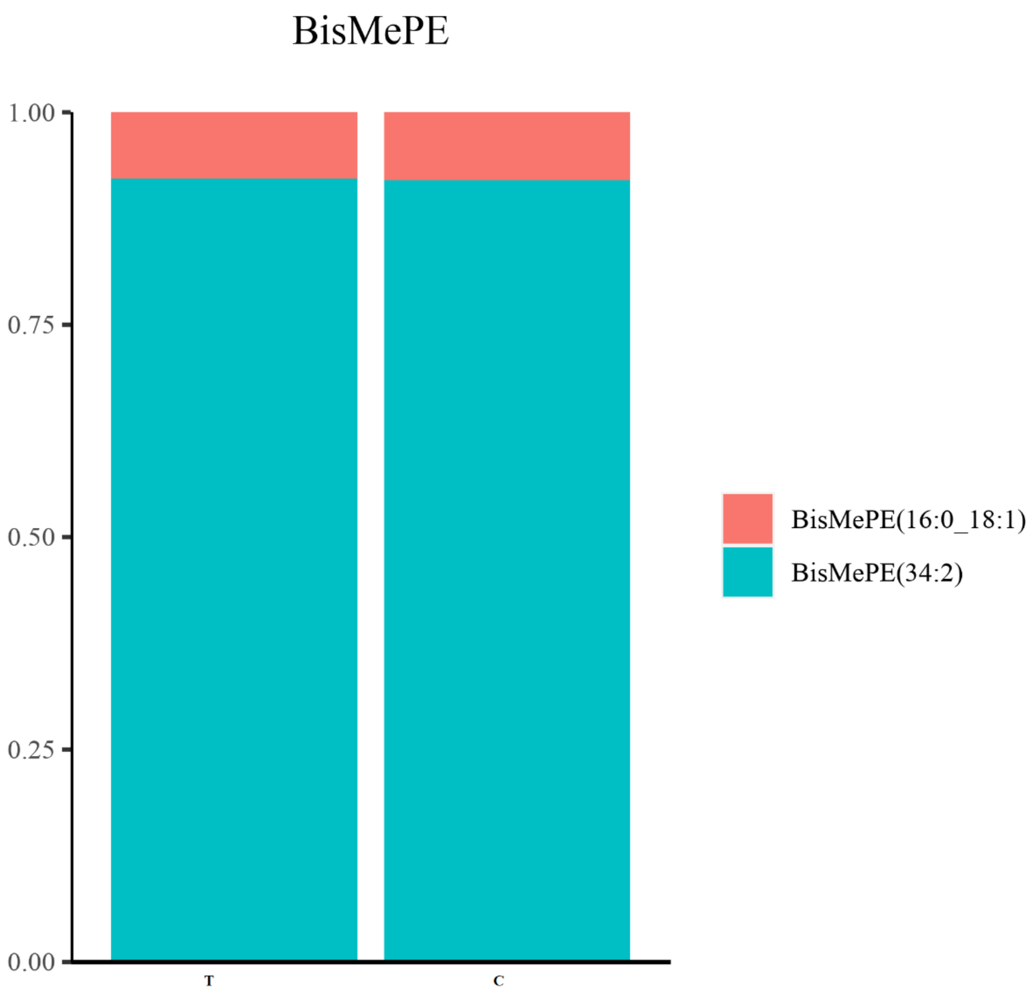

Figure S8 Liver lipidome different lipids- BisMePE

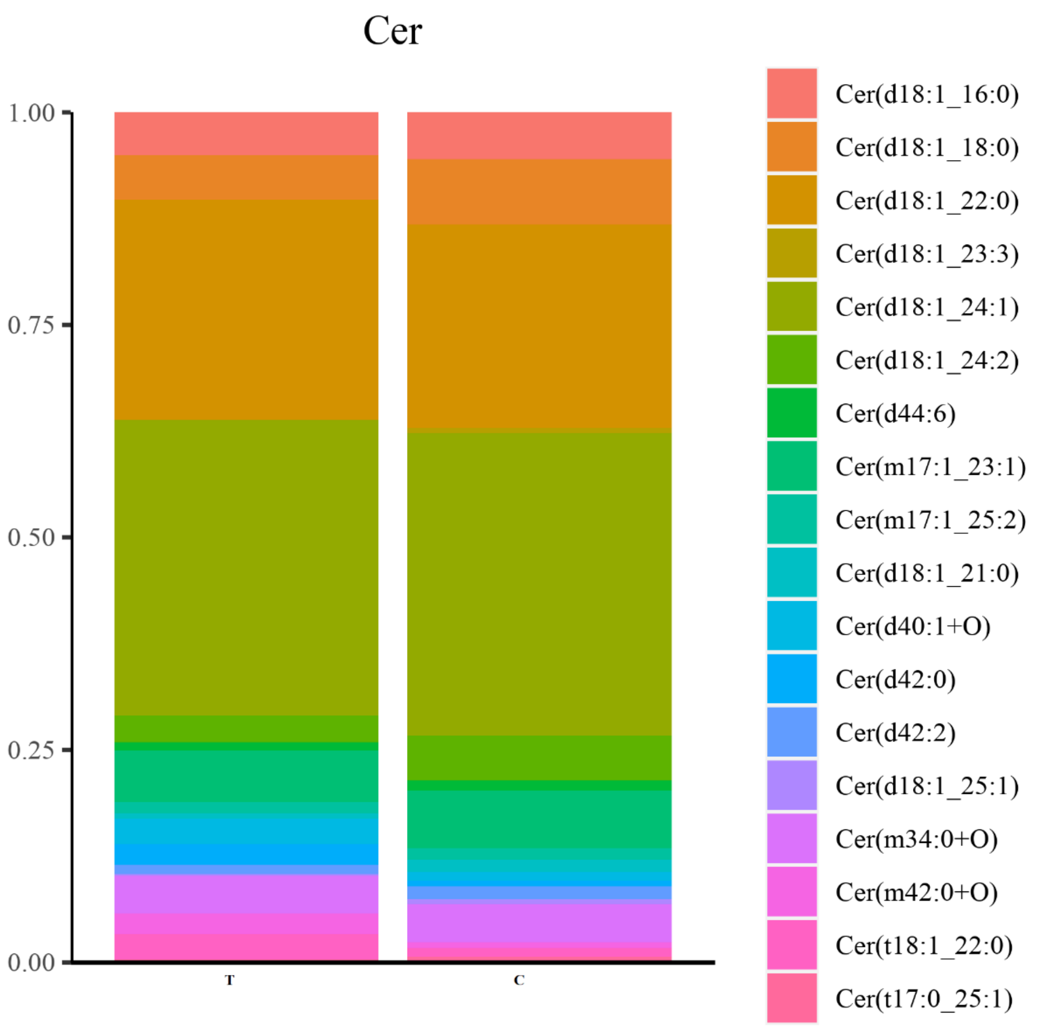

Figure S9 Liver lipidome different lipids-Cer

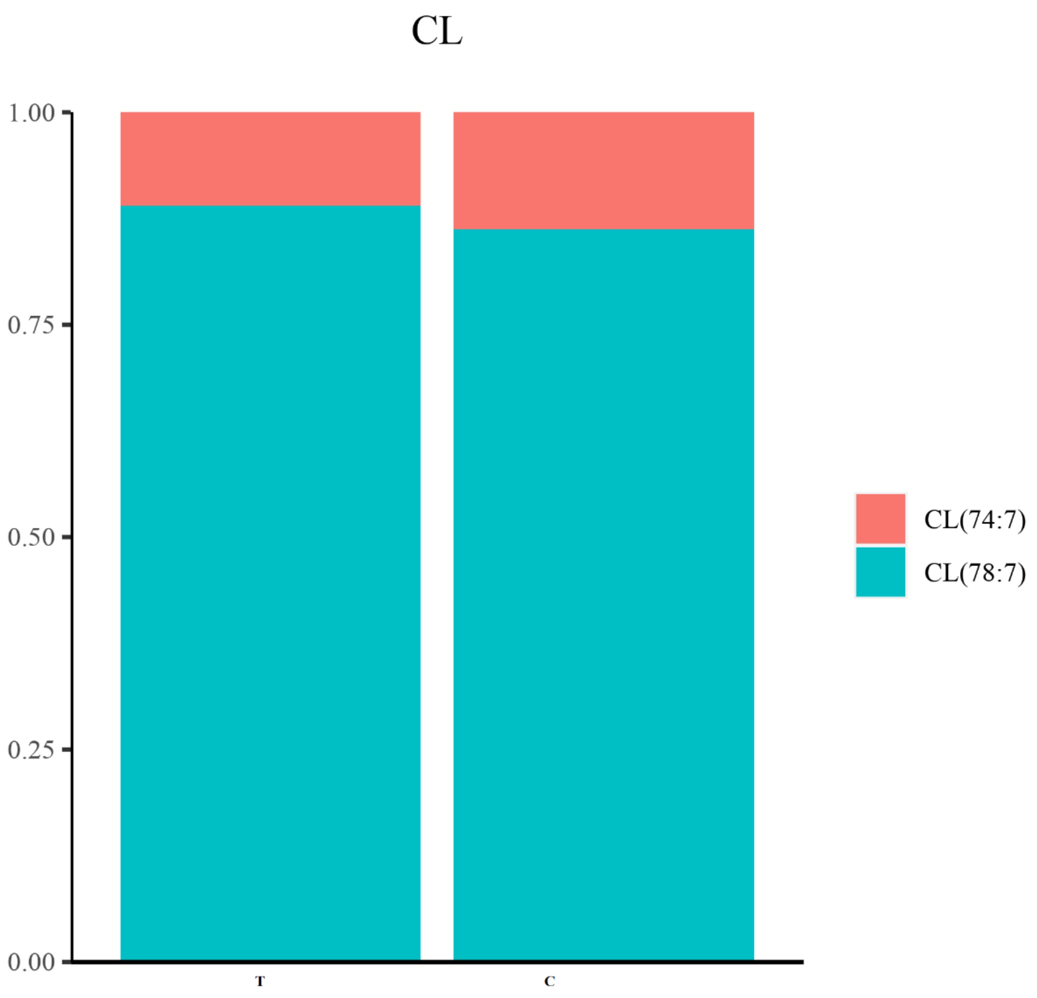

Figure S10 Liver lipidome different lipids-CL

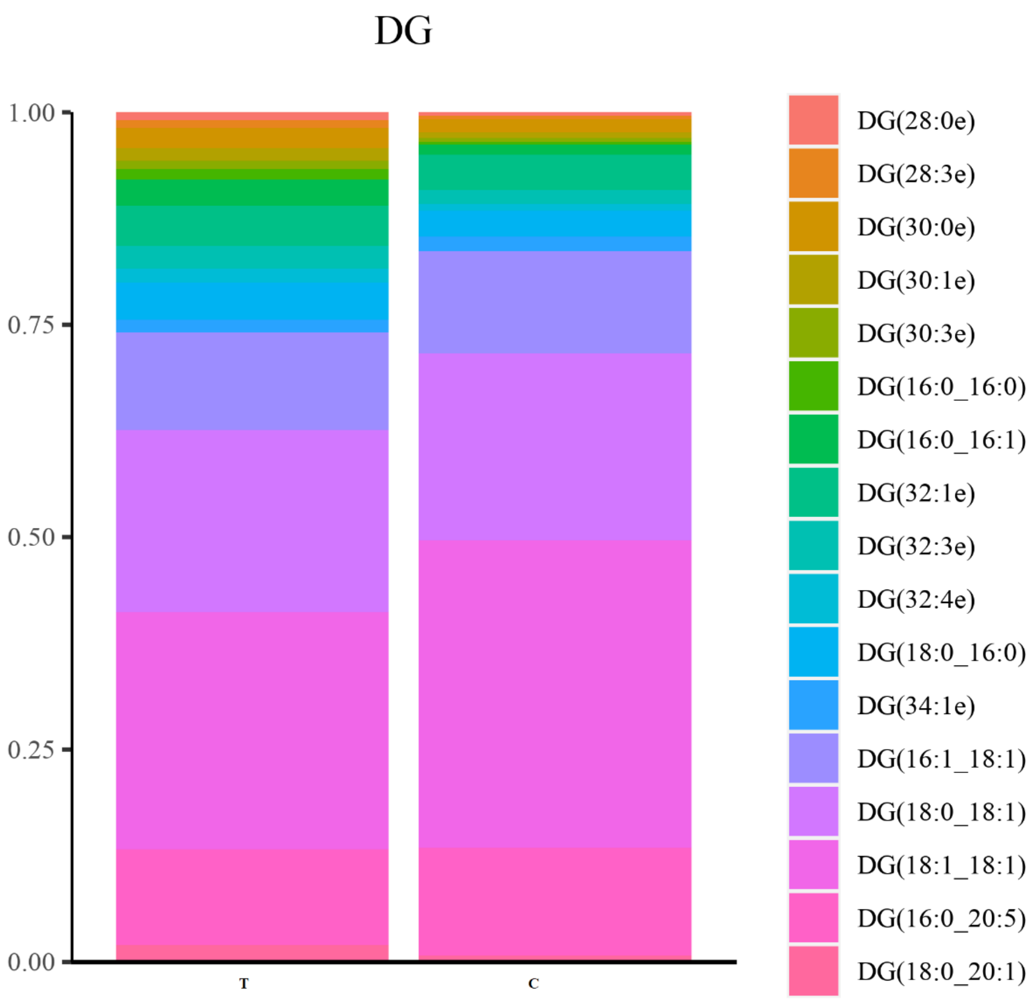

Figure S11 Liver lipidome different lipids-DG

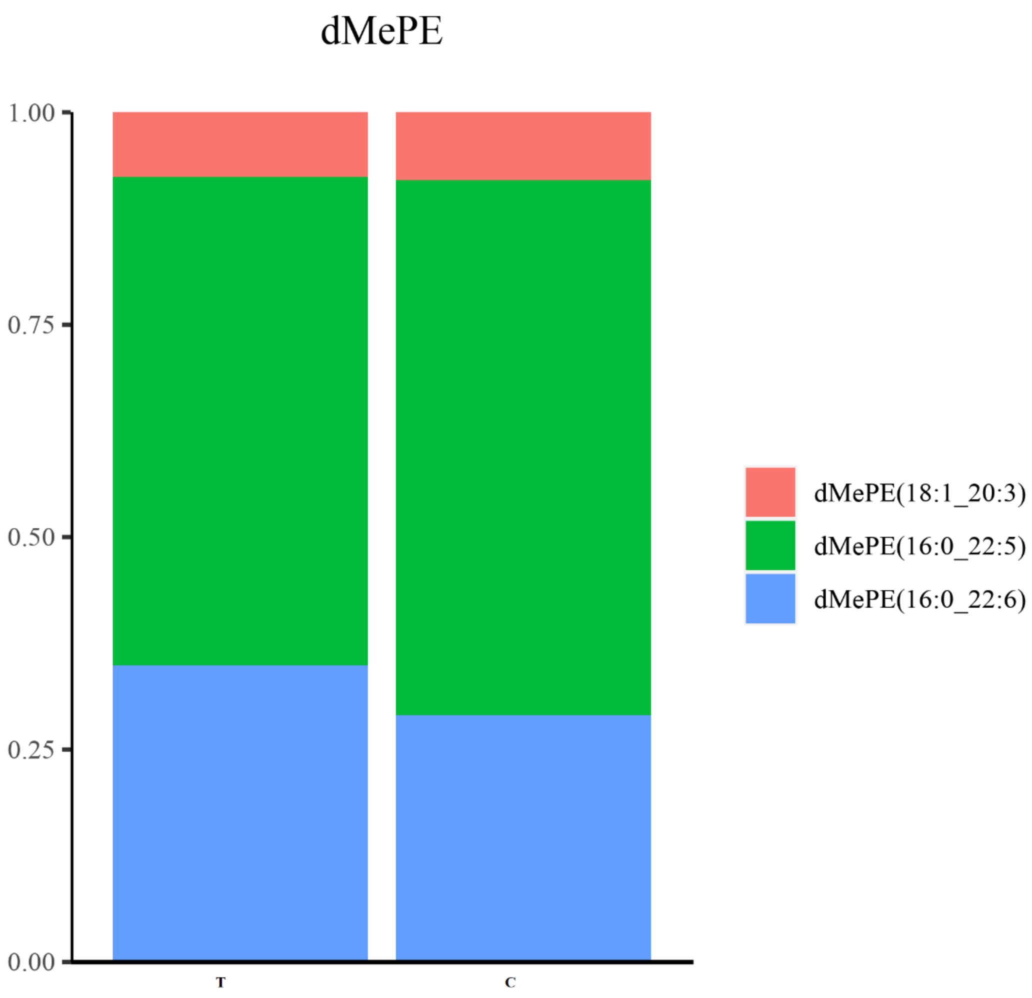

Figure S12 Liver lipidome different lipids-dMePE

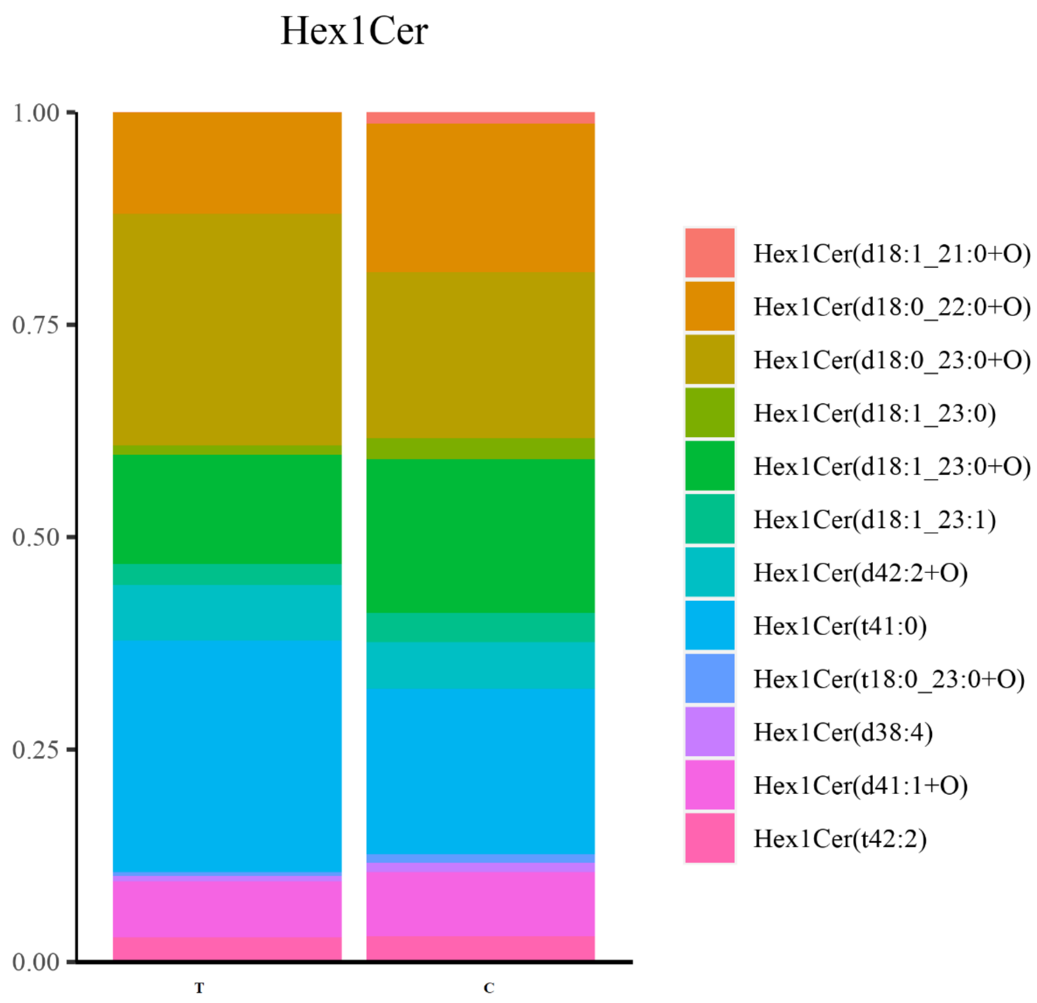

Figure S13 Liver lipidome different lipids- Hex1Cer

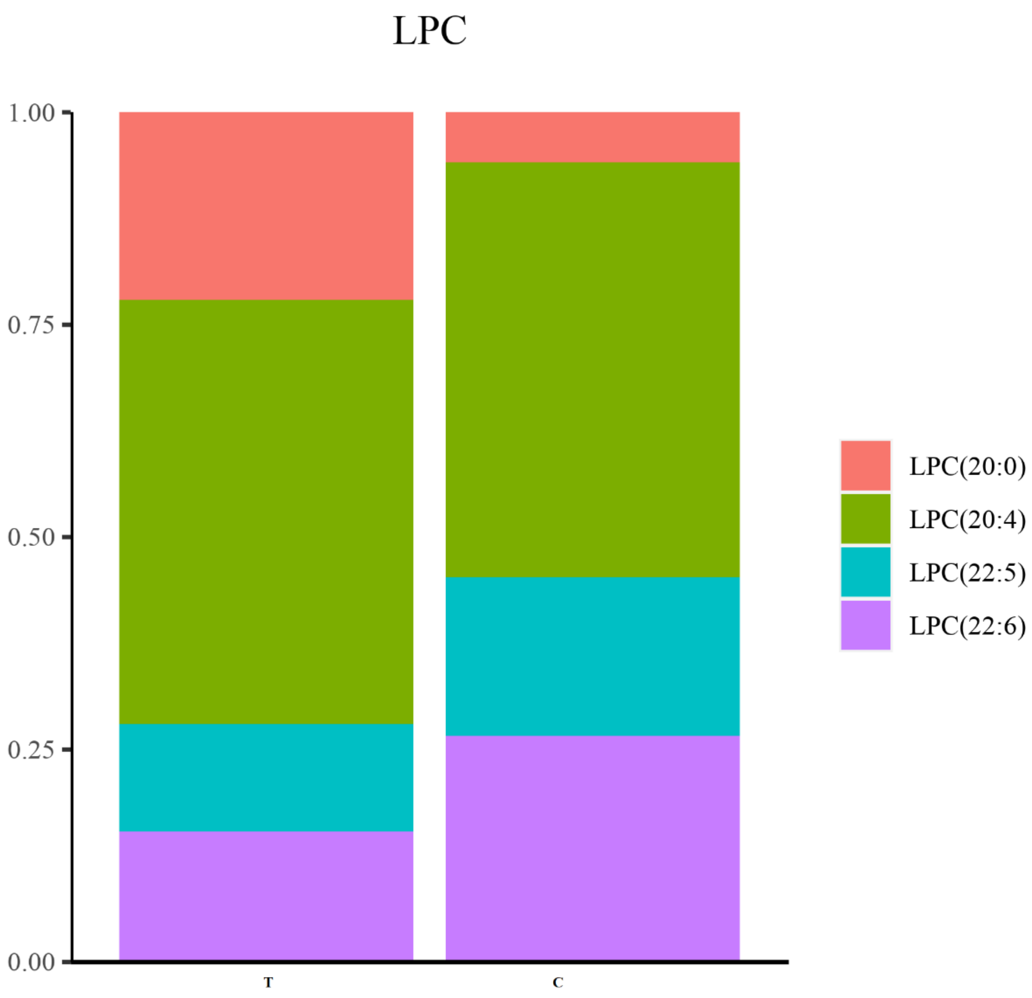

Figure S14 Liver lipidome different lipids-LPC

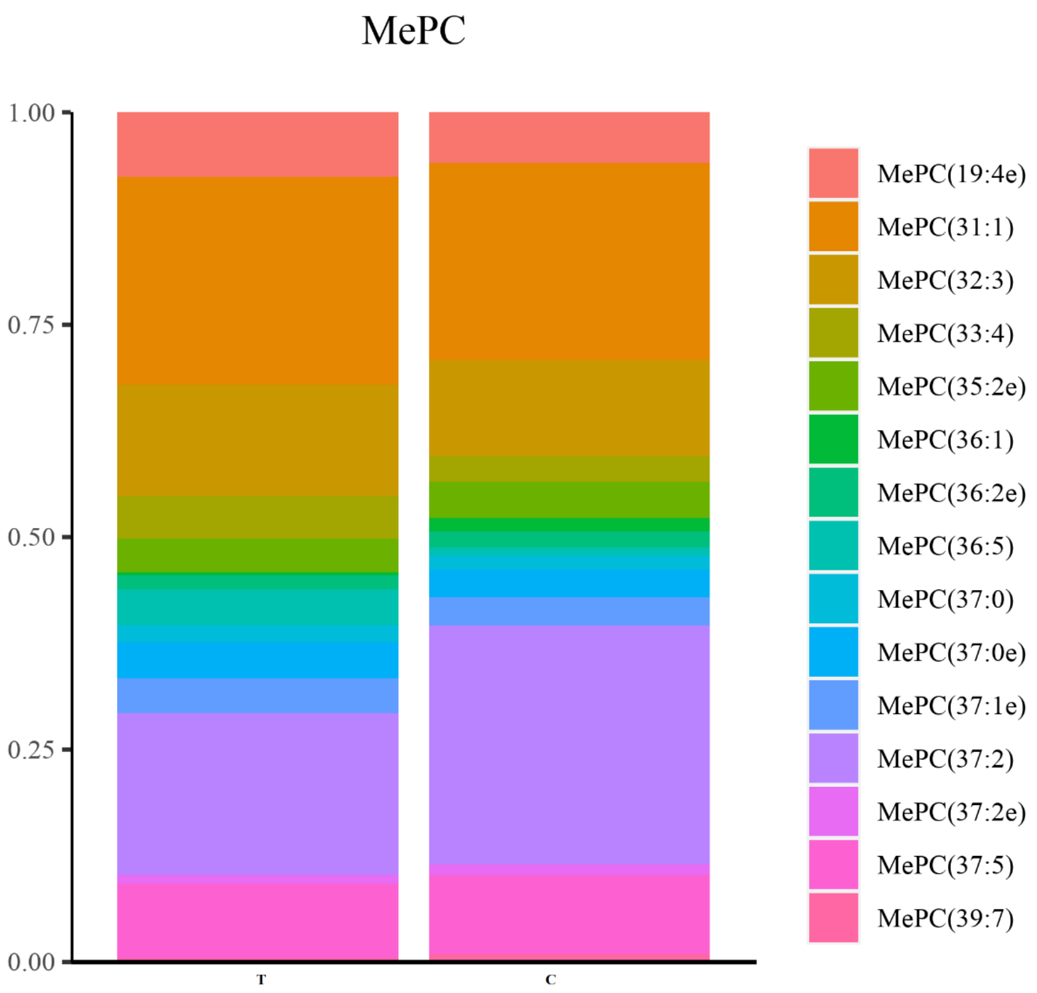

Figure S15 Liver lipidome different lipids-MePC

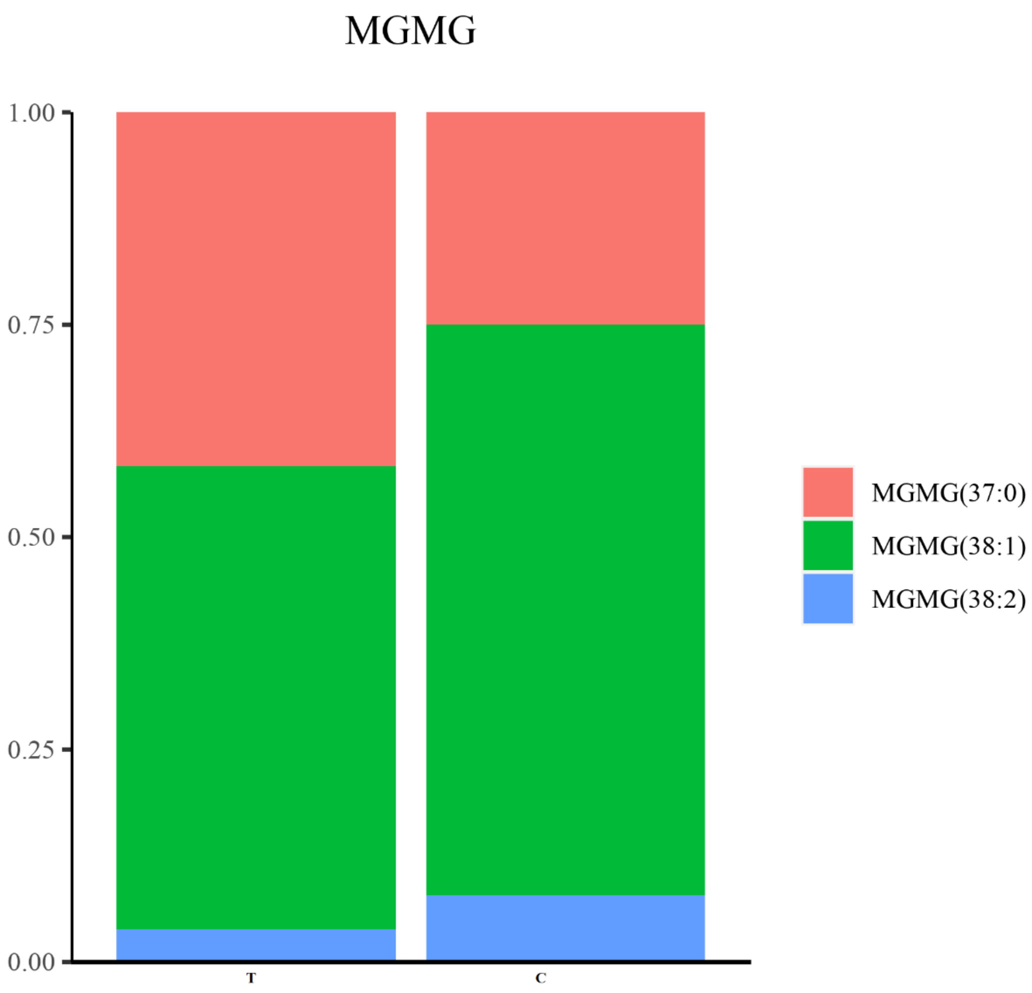

Figure S16 Liver lipidome different lipids-MGMG

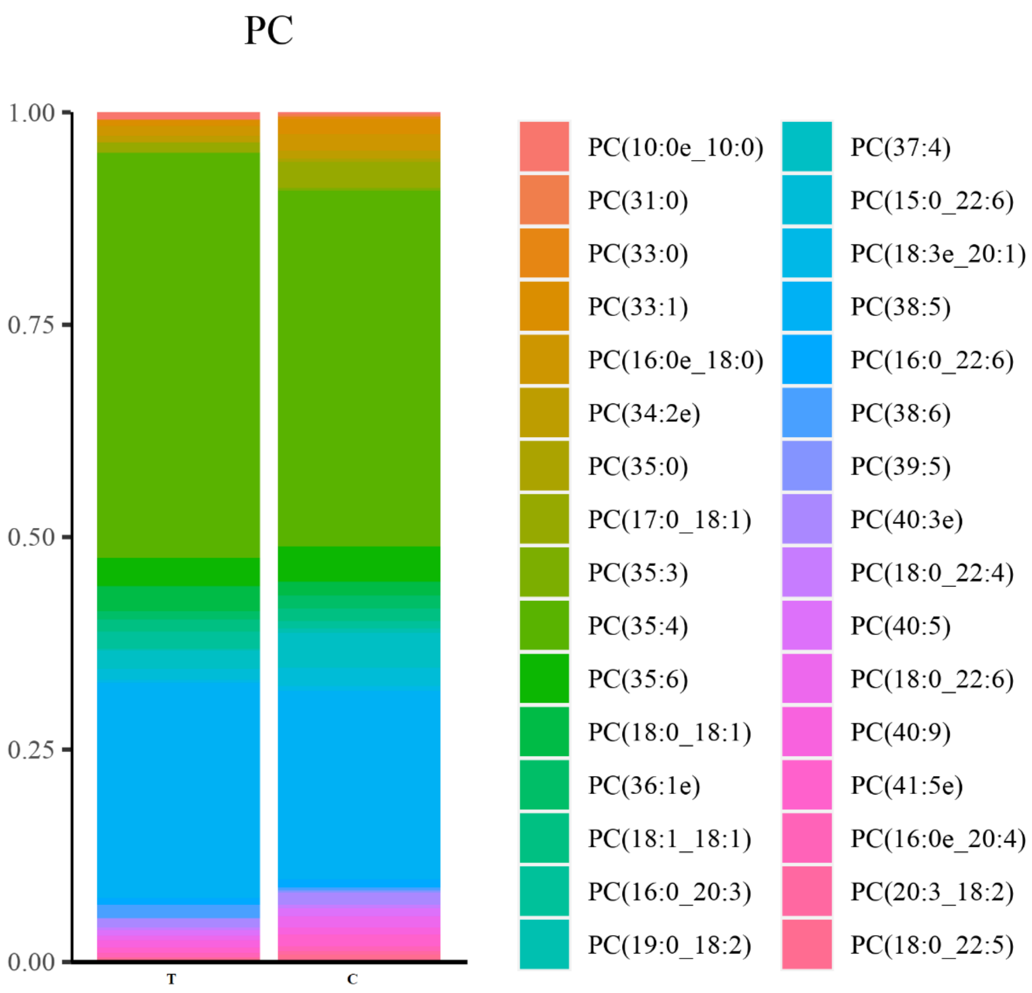

Figure S17 Liver lipidome different lipids-PC

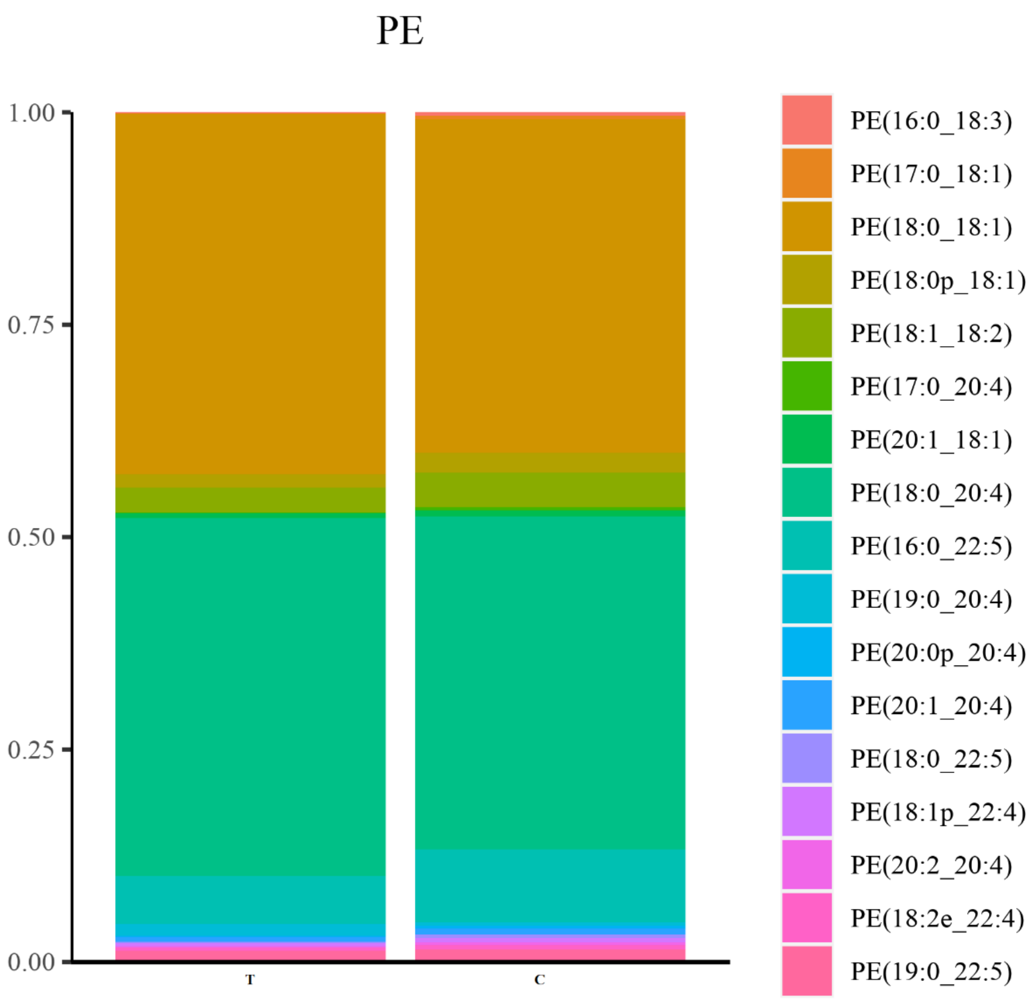

Figure S18 Liver lipidome different lipids-PE

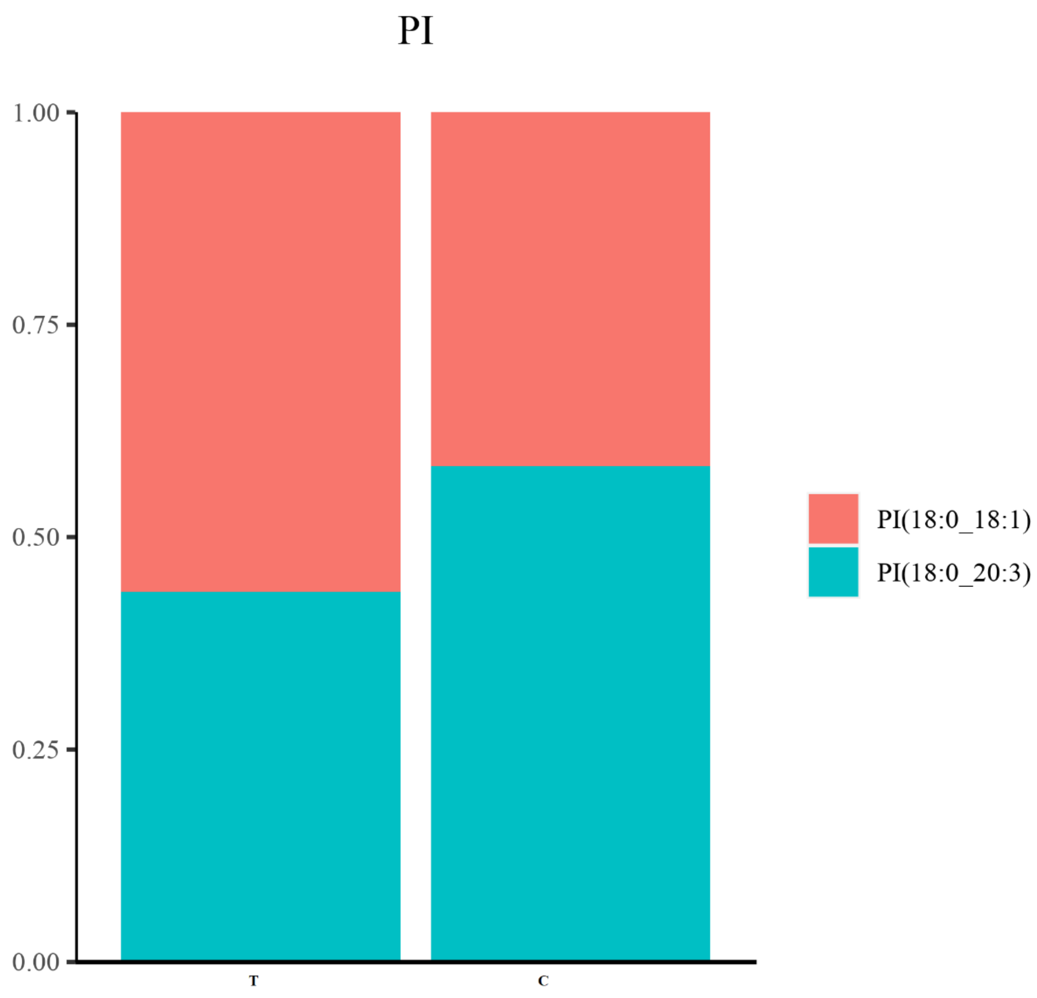

Figure S19 Liver lipidome different lipids-PI

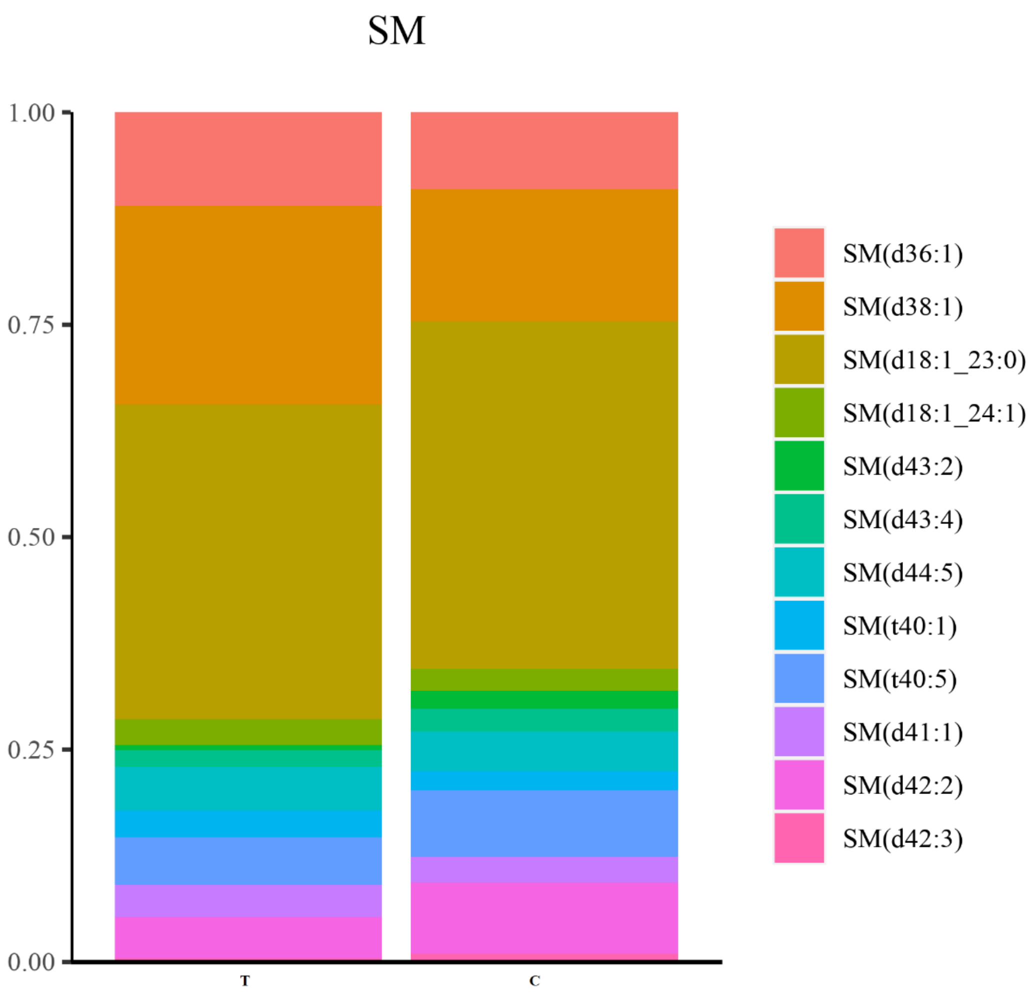

Figure S20 Liver lipidome different lipids-SM

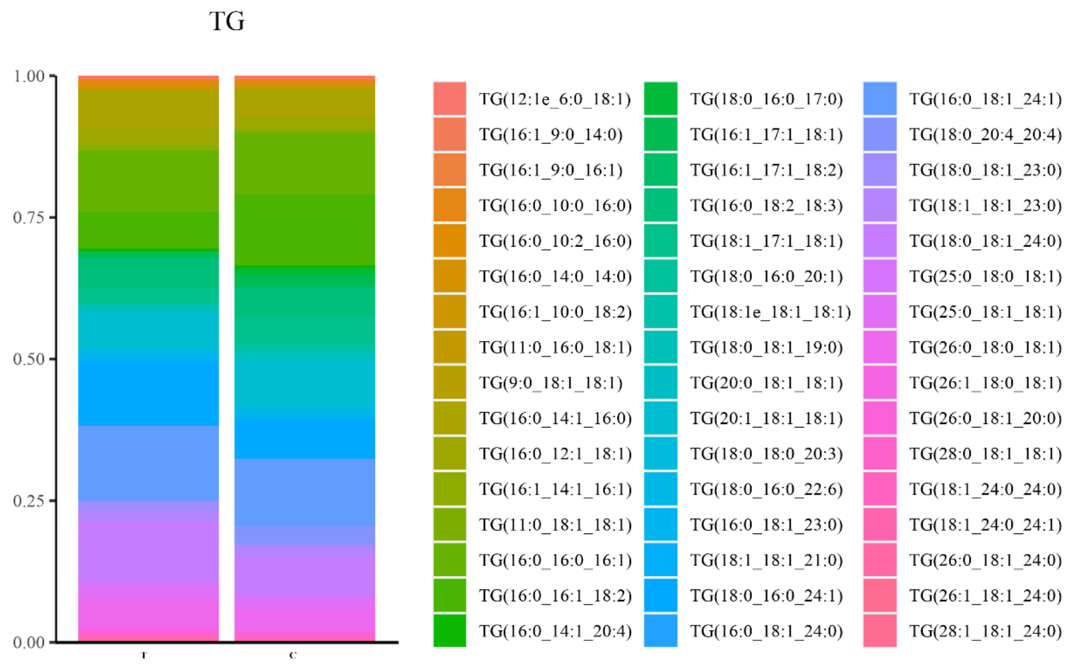

Figure S21 Liver lipidome different lipids-TG

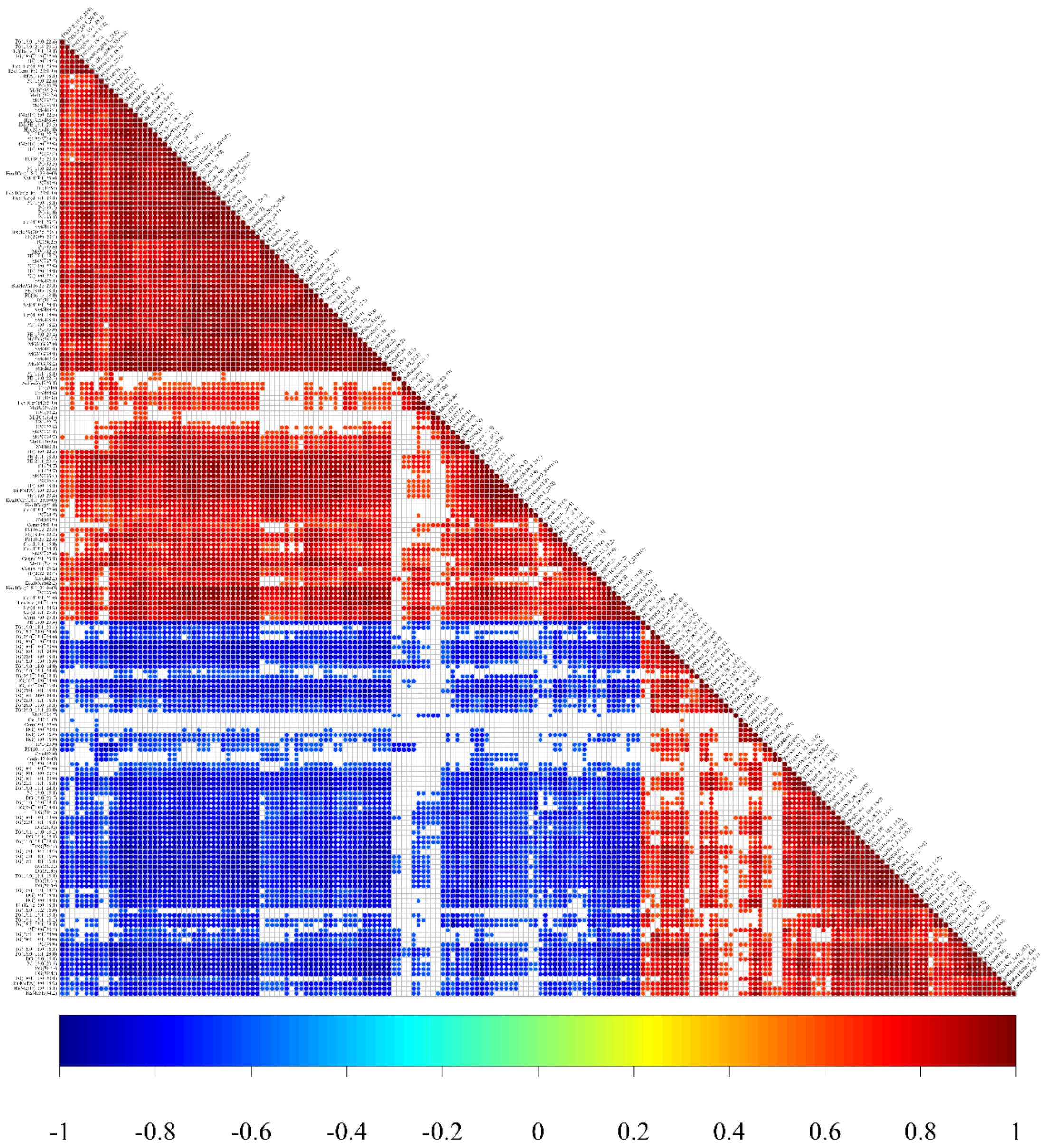

Figure S22 Correlation analysis between different lipids-heatmap
